# Individual and Combined Effects of Acute Caffeine and Sodium Bicarbonate Supplementation on Exercise Performance and Metabolic Responses: A Bayesian Network Meta-Analysis of Co-Supplementation Trials

**DOI:** 10.3390/metabo16090658

**Published:** 2026-09-08

**Authors:** Shenghui Liu, Xiaohan Fan, Tianyu Song, Jinfa Gu, Jian Zhu, Xing Zhang, Hansen Li, Hengzhi Deng

**Affiliations:** 1 Faculty of Sports and Exercise Science, Universiti Malaya, Jalan Universiti, Kuala Lumpur 50603, Malaysia; 2 School of Health Sciences, Universiti Sains Malaysia, Kubang Kerian 16150, Malaysia; 3 School of Physical Education, Huaqiao University, Quanzhou 362021, China; 4 Department of Physical Education and Sport, Faculty of Sport Sciences, University of Granada, 18071 Granada, Spain; 5 School of Physical Education, Sichuan Agricultural University, Ya’an 625014, China

**Keywords:** caffeine, sodium bicarbonate, co-ingestion, exercise performance, network meta-analysis

## Abstract

**Highlights:**

**What are the main findings?**

- Evidence from trials evaluating caffeine–sodium bicarbonate co-supplementation showed that caffeine, sodium bicarbonate, and their combination produced small but credible improvements in exercise performance relative to placebo, but co-ingestion was not clearly superior to either supplement alone.
- Co-ingestion elicited more pronounced metabolic responses, particularly increased blood lactate, without a proportionally greater improvement in exercise performance.

**What are the implications of the main findings?**

- Current evidence does not support the routine co-ingestion of caffeine and sodium bicarbonate solely to obtain an additional ergogenic benefit.
- Further adequately powered trials should examine standardized exercise contexts, co-ingestion dosing and timing strategies, and individual variation in responsiveness and tolerability.

**Abstract:**

**Background**: Caffeine and sodium bicarbonate (SB) are widely used ergogenic supplements that influence exercise performance through distinct physiological mechanisms and may differentially modulate exercise-related metabolic responses. Whether their co-ingestion provides benefits beyond either supplement alone remains uncertain. This study evaluated the incremental effects of caffeine and SB co-ingestion within trials specifically designed to investigate the combined supplementation strategy. **Methods**: A systematic review and Bayesian arm-based multilevel network meta-analysis of randomized controlled trials published up to May 2026 was conducted. Eligible studies investigated acute caffeine–SB co-supplementation and, where available, included caffeine-only and SB-only arms, with placebo as the common reference. Exercise performance was the primary outcome; blood lactate, blood pH, blood bicarbonate, and rating of perceived exertion were secondary outcomes. Effects were estimated using Hedges’ g, Bayesian credible intervals, and SUCRA rankings. **Results**: Thirteen studies involving 181 participants met the eligibility criteria. Within this co-supplementation trial network, caffeine (g = 0.21), SB (g = 0.21), and co-ingestion (g = 0.28) produced small but credible improvements in exercise performance relative to placebo. However, comparisons among active conditions provided no clear evidence that co-ingestion was superior to either supplement alone. SB increased blood pH (g = 0.95) and blood lactate (g = 1.13), whereas caffeine reduced blood bicarbonate (g = −0.89). Co-ingestion elicited the largest increase in blood lactate (g = 1.75), but this response was not accompanied by a proportionally greater improvement in performance. Exploratory analyses suggested possible variation in supplementation responses according to training status. **Conclusions**: Within trials designed to evaluate caffeine–SB co-supplementation, the caffeine-only, SB-only, and combined conditions showed small improvements in exercise performance relative to placebo. However, co-ingestion elicited more pronounced metabolic responses without a clear performance advantage over either component alone, and the mechanisms underlying this divergence remain uncertain.

## 1. Introduction

Caffeine (1,3,7-trimethylxanthine) is one of the most widely consumed and extensively studied ergogenic aids in sport and exercise science. A large body of evidence demonstrates that caffeine ingestion can enhance endurance performance, high-intensity exercise capacity, and cognitive function. These effects are primarily mediated through antagonism of adenosine receptors in the central nervous system, leading to reduced perception of effort, increased alertness, and enhanced motor unit recruitment [1,2]. In addition, caffeine may exert peripheral effects, including increased calcium release from the sarcoplasmic reticulum and altered substrate utilization, which may further contribute to improved performance [2].

In contrast, sodium bicarbonate (SB) acts predominantly as an extracellular buffering agent. Acute ingestion of SB elevates blood bicarbonate concentration and increases blood pH, thereby enhancing the body’s capacity to buffer hydrogen ions (H^+^) generated during high-intensity exercise. This buffering effect delays the development of metabolic acidosis and may facilitate the maintenance of glycolytic energy production, which is particularly beneficial during short-duration, high-intensity or repeated sprint activities [3,4].

Given their distinct physiological mechanisms, the co-ingestion of caffeine and SB has been proposed as a strategy to optimize exercise performance by targeting both central and peripheral determinants of fatigue. Specifically, caffeine may reduce perceived exertion and enhance central drive, while SB may improve acid–base balance and prolong the capacity for high-intensity work. This complementary interaction provides a strong theoretical basis for additive or even synergistic ergogenic effects.

However, the interaction between caffeine and SB is complex and may not always result in enhanced performance. For example, caffeine-induced increases in catecholamine release and glycolytic flux may accelerate lactate and hydrogen ion production, potentially increasing reliance on buffering systems. While this could theoretically augment the effectiveness of SB, it may also lead to earlier onset of fatigue if buffering capacity is exceeded. In addition, gastrointestinal (GI) discomfort, a well-documented side effect of SB ingestion, may be exacerbated by caffeine and negatively affect performance outcomes [4]. Furthermore, differences in supplementation timing, dosage, exercise modality, and participant characteristics may all influence the effectiveness of combined supplementation.

To date, studies investigating the combined effects of caffeine and SB have reported inconsistent findings. Some studies have demonstrated improved performance following co-ingestion compared with placebo [5,6], whereas others have shown no clear advantage over caffeine alone [7,8,9]. This inconsistency highlights an important unresolved question: whether any observed benefits reflect a true additive or synergistic interaction between caffeine and SB, or are primarily driven by one of the individual supplements. Clarifying this distinction is essential for both mechanistic understanding and the practical optimization of supplementation strategies in sport.

Therefore, the present systematic review and network meta-analysis focused on randomized trials investigating acute caffeine and SB co-supplementation. Using placebo as the common reference condition, co-ingestion was compared with the corresponding caffeine-only and SB-only conditions available within eligible trials. The primary objectives were to evaluate the effect of co-ingestion on exercise performance relative to placebo and to assess whether it provided an incremental benefit over either supplement alone. Secondary objectives were to compare the associated metabolic and perceptual responses and to explore potential variation in supplementation effects according to participant and intervention characteristics.

## 2. Methods

This systematic review and network meta-analysis was pre-registered on the Open Science Framework (OSF) on 9 March 2026 (Registration: osf.io/u6hr9) and conducted in accordance with the PRISMA 2020 statement and its extension for network meta-analyses [10]. The completed PRISMA checklist is available in Electronic Supplementary Material S1.

### 2.1. Eligibility Criteria

This review followed the PICOs framework—(1) Participants: healthy adults (≥18 years); (2) Intervention: acute co-ingestion of caffeine and SB, with or without a prior loading protocol; (3) Comparators: placebo or control, together with caffeine-only and SB-only conditions when available within eligible trials; and (4) Outcomes: exercise performance as the primary outcome, with blood pH, blood bicarbonate, blood lactate concentration, and rating of perceived exertion (RPE) as secondary outcomes.

Studies were eligible if they: (1) were randomized controlled trials published in peer-reviewed journals; (2) examined the acute effects of combined caffeine and SB supplementation administered before and/or during exercise; (3) included healthy adults; (4) included a placebo or control condition; and (5) reported original experimental data in English. Studies were excluded if they: (1) did not include a caffeine–SB co-ingestion condition; (2) used exclusively chronic supplementation without an acute pre-exercise dose; (3) included other active ergogenic co-interventions that could independently affect performance or fatigue; (4) did not assess exercise performance; or (5) were non-original reports or provided insufficient methodological information.

Acute supplementation was defined a priori as caffeine and SB administered before and/or during a single exercise session, with outcomes assessed within the same trial. Studies incorporating a prior loading phase (e.g., multi-day SB loading) were eligible provided that an acute dose was administered before exercise and outcomes were assessed within the same session. Because the primary objective was to determine whether co-ingestion provided an incremental ergogenic benefit, eligible studies were required to include a caffeine–SB co-ingestion condition. This study-level eligibility requirement did not exclude caffeine-only or SB-only arms embedded within otherwise eligible trials; when available, these arms were extracted as component comparators, with placebo serving as the common reference condition. Only studies reporting at least one exercise-performance outcome were eligible for quantitative synthesis. Secondary physiological and perceptual outcomes were analyzed when available.

### 2.2. Data Sources and Search Strategy

The final systematic search was conducted on 1 May 2026, across PubMed, Web of Science, Cochrane Library, Embase, SciELO, and SPORTDiscus. The following Boolean search strategies were applied: (caffeine OR “1,3,7-trimethylxanthine”) AND (“sodium bicarbonate” OR bicarbonate OR NaHCO3 OR “baking soda”) AND (exercise OR “exercise performance” OR “physical performance” OR endurance OR strength OR power OR sprint OR “time trial”).

No date or filter restrictions were applied.

### 2.3. Data Extraction

All records were imported into Microsoft Excel and EndNote 21 for de-duplication. Two independent reviewers (S.H.L. and X.H.F.) screened titles, abstracts, and full texts, and subsequently extracted data from eligible studies. Any discrepancies were resolved through discussion and consensus.

The following data were extracted from each study: sample size, participant characteristics (age, sex, and training status), exercise modality and protocol, and study design. Detailed information on supplementation protocols was also recorded, including caffeine dose (mg·kg^−1^), SB dose (g·kg^−1^), timing of ingestion, and whether a prior loading strategy was applied. For studies with multiple intervention arms (e.g., caffeine alone, SB alone, combined supplementation, and placebo), data were extracted separately for each condition to enable comparative analyses.

The primary outcome was exercise performance, defined according to the main performance endpoint reported in each study (e.g., time trial performance, time to exhaustion, mean or peak power output, or total work performed). When multiple performance outcomes were reported, the most ecologically valid and commonly used metric within the study was selected. Secondary outcomes included physiological and perceptual responses relevant to fatigue and metabolic stress, specifically blood pH, blood bicarbonate concentration, blood lactate concentration, and RPE. Where available, end-exercise values or values obtained at comparable time points were extracted to enhance consistency across studies.

When data were not reported numerically, authors were contacted or WebPlotDigitizer (v4.8) was used for extraction [11].

### 2.4. Quality and Risk of Bias Assessment

Methodological quality was assessed using a modified Physiotherapy Evidence Database (PEDro) scale, incorporating an additional item evaluating the effectiveness of blinding to the placebo condition [12]. Total scores ranged from 0 to 11 and were categorized as excellent (10–11), good (7–9), fair (5–6), or poor (<5). Two reviewers (T.Y.S. and S.H.L.) independently conducted the assessments, with discrepancies resolved through discussion or consultation with a third reviewer (H.Z.D.).

Risk of bias was evaluated using the Cochrane Risk of Bias 2 (RoB 2) tool. For crossover trials, the adapted RoB 2 version was applied, including an additional domain assessing bias arising from period and carryover effects [13]. The following domains were evaluated: randomization process, deviations from intended interventions, missing outcome data, outcome measurement, selection of the reported result, and period/carryover effects (where applicable). Assessments were performed independently by the same reviewers, with disagreements resolved by consensus.

### 2.5. Statistical Analysis

#### 2.5.1. Effect Size Calculation and Data Synthesis

All effect size calculations followed the Cochrane Handbook for Systematic Reviews of Interventions (Version 6.5, 2024) [14]. Given the relatively small sample sizes of the included studies, Hedges’ g was used to estimate standardized mean differences (SMDs) between intervention and comparator conditions, with correction for small-sample bias [15].

Because both crossover and parallel designs were included, effect sizes were calculated according to study design. For crossover trials, the paired structure was accounted for by incorporating the within-participant correlation (r) when deriving SMDs, using change scores when available. For parallel trials, SMDs were calculated from between-group differences based on post-intervention values or change scores as reported.

As most studies did not report correlation coefficients, a value of r = 0.50 was assumed for the primary analysis [16]. Sensitivity analyses were conducted using r = 0.20 and r = 0.80 to assess the robustness of the findings [17].

Effect sizes (g) were interpreted using conventional thresholds: trivial (<0.2), small (0.2–0.5), medium (0.5–0.8), and large (>0.8). Detailed computational formulas and step-by-step procedures are provided in Supplementary Material S2.

#### 2.5.2. Network Meta-Analysis

A Bayesian arm-based multilevel network meta-analysis was conducted to estimate the effects of caffeine, SB, and their co-ingestion, with placebo specified as the common reference condition. The primary comparison of interest was whether co-ingestion provided an incremental benefit over either single-supplement condition. By modelling treatment effects at the arm level, this approach enabled comparisons among active conditions within a unified framework while accounting for their shared trial context [18].

All analyses were performed in RStudio (version 2024.09.1, Build 394) using the *brms* package. To account for the dependency arising from multiple effect sizes within the same study, a multilevel structure was specified with effects nested within studies. The Bayesian framework provides posterior distributions of treatment effects, reported as means with 95% credible intervals (CrIs), reflecting the uncertainty around the estimates [19].

The effect of each active condition was initially estimated relative to the common placebo reference. Contrasts among active conditions, including caffeine versus SB and co-ingestion versus either component alone, were subsequently derived from the fitted model using the *emmeans* package and reported with 95% highest posterior density (HPD) intervals [20]. These estimates therefore characterized the relative effects of the active conditions within the eligible co-supplementation trial network.

Treatment ranking was derived from posterior ranking probabilities obtained from the Bayesian model. SUCRA values were calculated based on these probabilities to summarize the relative effectiveness of each intervention. SUCRA values range from 0 to 1, with higher values indicating a greater likelihood of being the most effective treatment [21].

Prediction intervals (PIs) were calculated to evaluate the expected range of true effects in future studies by incorporating both parameter uncertainty and between-study heterogeneity (τ^2^). This was achieved by simulating effects from the posterior distribution of the hierarchical model [22,23].

To aid interpretation of practical relevance, a small-effect threshold was predefined as an absolute standardized mean difference (SMD) of 0.20, in accordance with conventional benchmarks for small effect sizes [24]. Estimated effects were evaluated against direction-specific thresholds of +0.20 or −0.20, and the posterior probabilities of exceeding the positive threshold or falling below the negative threshold were calculated to provide a probabilistic interpretation of practical significance [25].

#### 2.5.3. Exploratory Subgroup Analyses

To explore potential sources of heterogeneity and identify possible variation in the effects of caffeine, SB, and their co-ingestion across study characteristics, exploratory subgroup analyses were conducted for the primary outcome. The following study-level characteristics were examined:

**(1)**  **Training status:** Participants were categorized into two groups (recreationally active vs. trained/athlete) based on established participant classification frameworks [26]. Recreationally active individuals were defined as participants engaging in regular physical activity without objective performance or fitness criteria, whereas the trained/athlete group included individuals with structured training backgrounds, objective physiological indicators (e.g., VO_2_max/VO_2_peak), or explicit classification as trained or competitive athletes. When discrepancies occurred between descriptive terminology and physiological characteristics, objective indicators were prioritized.
**(2)**  **Exercise task type**: Exercise outcomes were categorized a priori into two physiologically meaningful domains based on predominant metabolic demand and mechanistic relevance to SB (i.e., enhancement of extracellular buffering capacity). These domains are:
  (i) High-intensity endurance/glycolytic performance, including outcomes characterized by substantial hydrogen ion accumulation and susceptibility to acidosis-related fatigue. This category encompassed time-trial performance, time-to-exhaustion, repeated-sprint ability, sport-specific exhaustive tasks, and muscular endurance (e.g., repetitions to failure at submaximal loads).
  (ii) Maximal/explosive neuromuscular performance, including outcomes primarily reflecting peak force or power during brief, single-effort tasks with minimal contribution from cumulative metabolic acidosis. This category encompassed maximal voluntary force, handgrip strength, jump performance, and peak power output.

This classification was selected to align with the proposed mechanism of SB while avoiding excessive subgroup fragmentation given the limited number of included studies. Although muscular endurance lies on a continuum of fatigue mechanisms, its predominant limitation by acidosis supports its inclusion within the high-intensity glycolytic domain.

**(3)**  **Types of supplements:** Interventions were categorized into solid (e.g., capsules or tablets) and liquid (e.g., beverages or solutions) forms based on the mode of administration. This classification was applied to account for potential differences in absorption kinetics and gastrointestinal tolerance between delivery forms.

Given the limited and unevenly distributed data, subgroup analyses were considered exploratory. For each study characteristic, subgroup-specific treatment effects relative to placebo and pairwise contrasts among the active conditions were estimated from the fitted model using the *emmeans* package and reported with 95% HPD intervals. Analyses were restricted to exercise performance, as data for secondary outcomes, supplement timing, and dosage were insufficient or heterogeneously reported.

#### 2.5.4. Publication Bias and Sensitivity Analyses

Publication bias was assessed using contour-enhanced funnel plots and multilevel Egger regression tests [27,28]. In addition, the S-value was calculated to quantify the extent of publication bias required to attenuate the observed effects, with higher values indicating greater robustness.

Because all effect sizes were calculated relative to a common comparator (placebo), the resulting data structure did not preserve independent comparison pathways required for formal network inconsistency assessment within the primary Bayesian arm-based framework. To evaluate the robustness of comparisons among active interventions, a supplementary contrast-based network meta-analysis was conducted using the *netmeta* R package. Within this framework, global inconsistency was explored using the design-by-treatment interaction model, and local inconsistency was assessed using node-splitting analyses where applicable and supported by the available data structure. These analyses were performed as sensitivity analyses to examine whether the comparative results and treatment rankings were robust across different analytical approaches, rather than as formal inconsistency assessments of the primary model.

## 3. Results

### 3.1. Studies Retrieved

**Figure 1:**
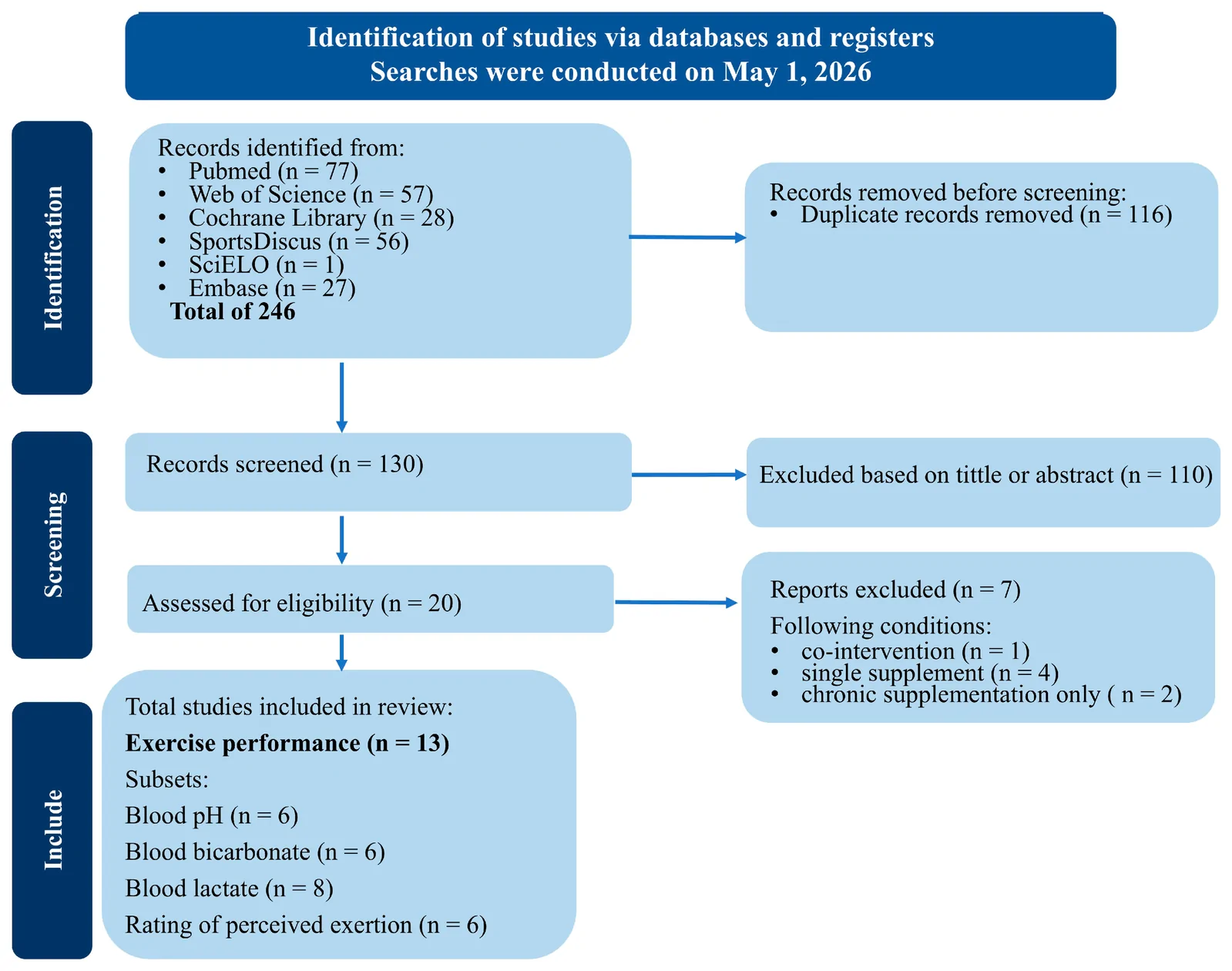
PRISMA flow diagram for included and excluded studies.

The initial database search identified 246 records. Following title, abstract, and full-text screening, 13 studies met the inclusion criteria and assessed exercise performance, contributing a total of 30 effect size estimates. Additionally, 6 studies reported RPE, 8 reported blood lactate, and 6 studies each reported blood bicarbonate and blood pH (Figure 1).

### 3.2. Characteristics of Included Studies

In total, 181 participants were included across the 13 studies, comprising 128 men and 35 women, while two studies (n = 18) did not report participant sex. Sample sizes ranged from 6 to 28 participants. Most studies recruited male-only samples, whereas a smaller number included mixed-sex cohorts, and no study specifically examined female-only populations. All included studies employed randomized, double-blind crossover designs. Most studies investigated acute supplementation protocols involving caffeine, SB, and their co-ingestion, with the exception of one study that did not include a caffeine-only condition [29].

Participants were predominantly trained or recreationally active individuals, with only a minority of studies involving elite athletes [7,30]. Exercise tasks were heterogeneous and included cycling time trials or time-to-exhaustion tests, rowing performance, repeated sprint protocols, resistance exercise, and sport-specific tests such as judo and karate, reflecting a broad range of physiological and neuromuscular demands.

Regarding outcome reporting, RPE was assessed in six studies, representing a total of 73 participants. Blood-based physiological markers were inconsistently reported: blood bicarbonate and pH were measured in six studies (n = 59), while blood lactate was reported in eight studies (n = 90).

Supplementation protocols varied across studies. Caffeine was typically administered at doses of 3–6 mg·kg^−1^ approximately 30–60 min prior to exercise, whereas SB was commonly provided at ~0.3 g·kg^−1^, either as a single bolus 60–100 min before exercise or in split doses over 60–120 min to improve tolerability. In several studies, bicarbonate ingestion followed multi-timepoint strategies (e.g., −120, −90, and −60 min) or short-term loading phases over 1–3 days to reduce gastrointestinal discomfort [6,31]. With the exception of three studies [32,33,34], combined supplementation protocols generally involved staggered ingestion, with caffeine administered closer to exercise onset and bicarbonate consumed earlier to allow sufficient time for alkalosis development. Regarding supplement formulation, most studies employed solid forms, such as capsules or pills, whereas five studies used liquid-based delivery methods, including solutions or beverages [8,32,33,34,35]. For more details, please refer to Table 1.

### 3.3. Adverse Effects Summary

Adverse effects and tolerability were reported in the majority of included studies, primarily focusing on GI symptoms associated with SB ingestion. Most studies employed subjective assessments, including GI symptom questionnaires, abdominal discomfort scales, or stomach comfort ratings, to evaluate tolerability. Across studies, SB, either alone or combined with caffeine, was consistently associated with increased GI-related symptoms compared with placebo or caffeine-only conditions, including nausea, diarrhea, stomach discomfort, dizziness, and flatulence [3,8,30,31,32,34,35]. In contrast, caffeine alone was generally well tolerated and not associated with meaningful increases in GI symptoms, although occasional mild effects (e.g., jitteriness or discomfort) were reported [7,30].

However, these symptoms were generally mild to moderate in severity and transient in nature, with most participants able to complete the exercise protocols without performance limitation [29,31,34]. Notably, co-ingestion of caffeine with SB did not consistently exacerbate GI symptoms beyond bicarbonate alone, suggesting that adverse effects are primarily driven by SB rather than caffeine per se [8,32,35].

Importantly, several studies reported minimal or no significant GI disturbances, particularly when symptom severity was low or comparable across conditions, indicating that tolerability may depend on dosing strategies and ingestion protocols [6,7,9]. In contrast, only one study did not report any adverse-effect or tolerability outcomes [33]. Overall, while GI symptoms are a common side effect of SB supplementation, their severity is typically insufficient to impair exercise performance under controlled experimental conditions. For more details, please refer to Supplementary Material S3.

### 3.4. Primary Analysis

#### 3.4.1. Exercise Performance

**Figure 2:**
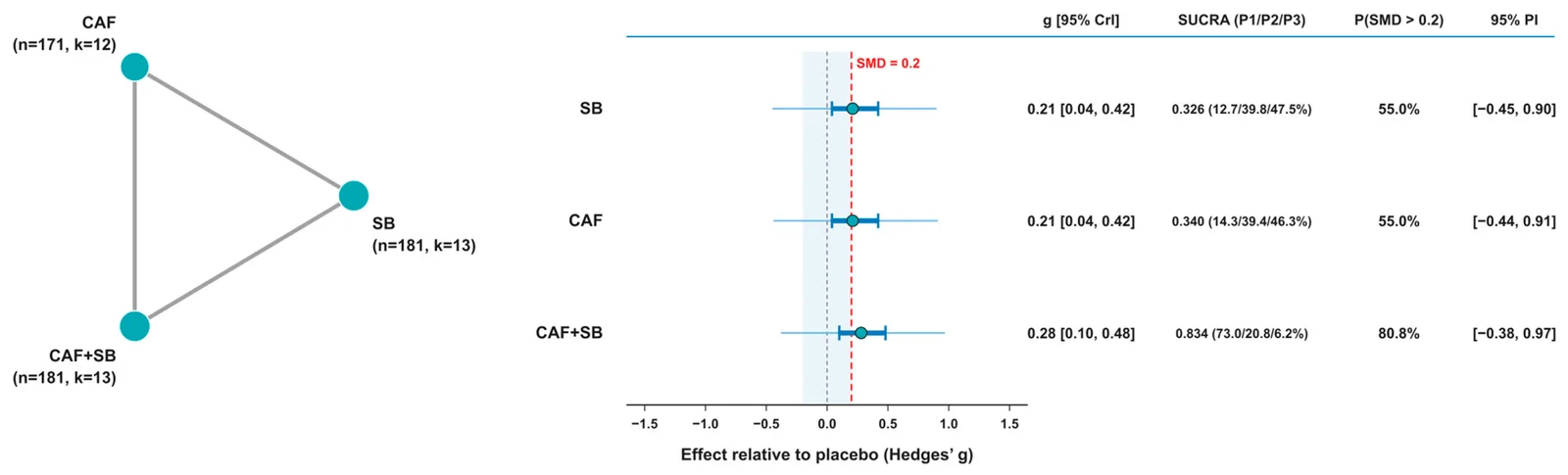
Network geometry and treatment effects on exercise performance. Notes: CAF: Caffeine; SB: Sodium bicarbonate; CAF + SB: Caffeine and sodium bicarbonate co-ingestion; CrI: Credible interval; PI: Prediction interval; SUCRA: Surface under the cumulative ranking curve; k: Number of included studies; n: Number of participants. Effects are expressed as standardized mean differences (Hedges’ g) relative to placebo. CAF and SB estimates were derived exclusively from single-supplement arms within eligible co-supplementation trials and do not represent their broader supplement-specific evidence bases. The vertical dashed line indicates no effect (g = 0), and the shaded region represents a predefined small-effect threshold (g = 0.20). P(SMD > 0.20) denotes the posterior probability of exceeding this threshold. SUCRA values reflect the probability of each treatment ranking best, second, or third. Node sizes are proportional to sample size, and edge thickness reflects the number of direct comparisons.

Within the network of eligible co-supplementation trials, SB (k = 13, g = 0.21, 95% CrI [0.04, 0.42], PI [−0.45, 0.90]), caffeine (k = 12, g = 0.21, 95% CrI [0.04, 0.42], PI [−0.44, 0.91]), and the combined condition (k = 13, g = 0.28, 95% CrI [0.10, 0.48], PI [−0.38, 0.97]) each showed small credible improvements relative to placebo. No meaningful differences were observed among the active conditions based on posterior contrasts (all HPD intervals crossed zero), providing no clear evidence that co-ingestion conferred an incremental performance benefit over caffeine or SB alone. The combined condition had the highest SUCRA value (0.83), followed by caffeine (0.34) and SB (0.33); however, rankings were interpreted cautiously in the absence of credible active-treatment differences. The three active conditions exceeded the predefined small-effect threshold (g = 0.20) within this restricted network (Figure 2; see also Supplementary Material S4 for the pairwise comparisons plot).

#### 3.4.2. Rating of Perceived Exertion

**Figure 3:**
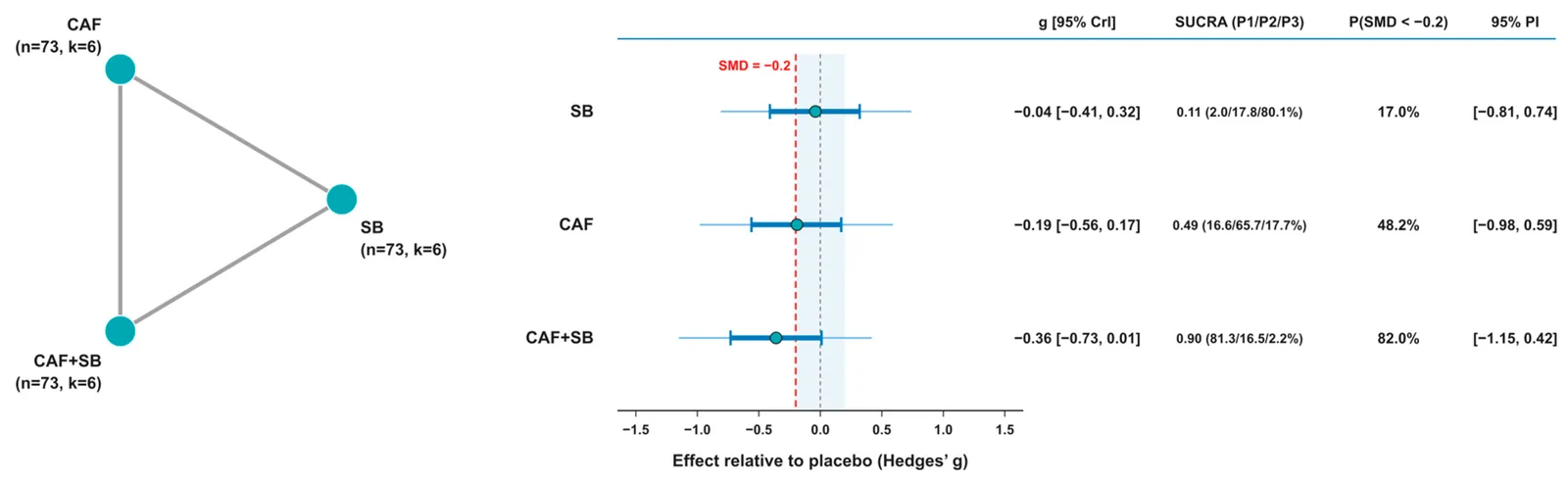
Network geometry and treatment effects on rating of perceived exertion. Notes: CAF: Caffeine; SB: Sodium bicarbonate; CAF + SB: Caffeine and sodium bicarbonate co-ingestion; CrI: Credible interval; PI: Prediction interval; SUCRA: Surface under the cumulative ranking curve; k: Number of included studies; n: Number of participants. Effects are expressed as standardized mean differences (Hedges’ g) relative to placebo. CAF and SB estimates were derived exclusively from single-supplement arms within eligible co-supplementation trials and do not represent their broader supplement-specific evidence bases. The vertical dashed line indicates no effect (g = 0), and the shaded region represents a predefined small-effect threshold (g = −0.20). P(SMD < −0.20) denotes the posterior probability of falling below this threshold. SUCRA values reflect the probability of each treatment ranking best, second, or third. Node sizes are proportional to sample size, and edge thickness reflects the number of direct comparisons.

For RPE, none of the active conditions showed a credible reduction relative to placebo, as all CrIs crossed zero. Pairwise contrasts also showed no meaningful differences among active conditions. Although co-ingestion had the highest SUCRA value (0.90), its estimated effect was small and uncertain (g = −0.36, 95% CrI [−0.73, 0.01]); therefore, the ranking should be interpreted cautiously (Figure 3; see also Supplementary Material S4 for the pairwise comparisons plot).

#### 3.4.3. Blood Bicarbonate

**Figure 4:**
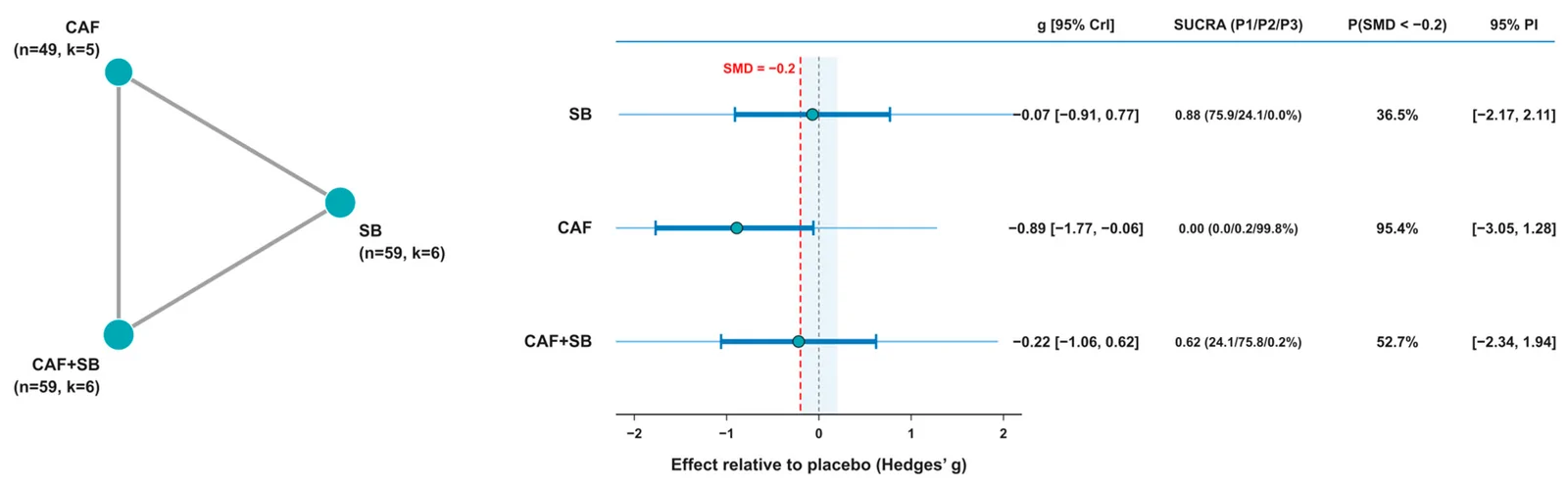
Network geometry and treatment effects on blood bicarbonate concentration. Notes: CAF: Caffeine; SB: Sodium bicarbonate; CAF + SB: Caffeine and sodium bicarbonate co-ingestion; CrI: Credible interval; PI: Prediction interval; SUCRA: Surface under the cumulative ranking curve; k: Number of included studies; n: Number of participants. Effects are expressed as standardized mean differences (Hedges’ g) relative to placebo. CAF and SB estimates were derived exclusively from single-supplement arms within eligible co-supplementation trials and do not represent their broader supplement-specific evidence bases. The vertical dashed line indicates no effect (g = 0), and the shaded region represents a predefined small-effect threshold (g = −0.20). P(SMD < −0.20) denotes the posterior probability of falling below this threshold. SUCRA values reflect the probability of each treatment ranking best, second, or third. Node sizes are proportional to sample size, and edge thickness reflects the number of direct comparisons.

For blood bicarbonate, caffeine produced a credible reduction relative to placebo (g = −0.89, 95% CrI [−1.77, −0.06]), whereas no clear effects were observed for SB or co-ingestion. Pairwise contrasts indicated lower blood bicarbonate following caffeine than following SB or co-ingestion. Consistent with these estimates, SB ranked highest for increasing blood bicarbonate (SUCRA = 0.88) (Figure 4; see also Supplementary Material S4 for the pairwise comparisons plot).

#### 3.4.4. Blood pH

**Figure 5:**
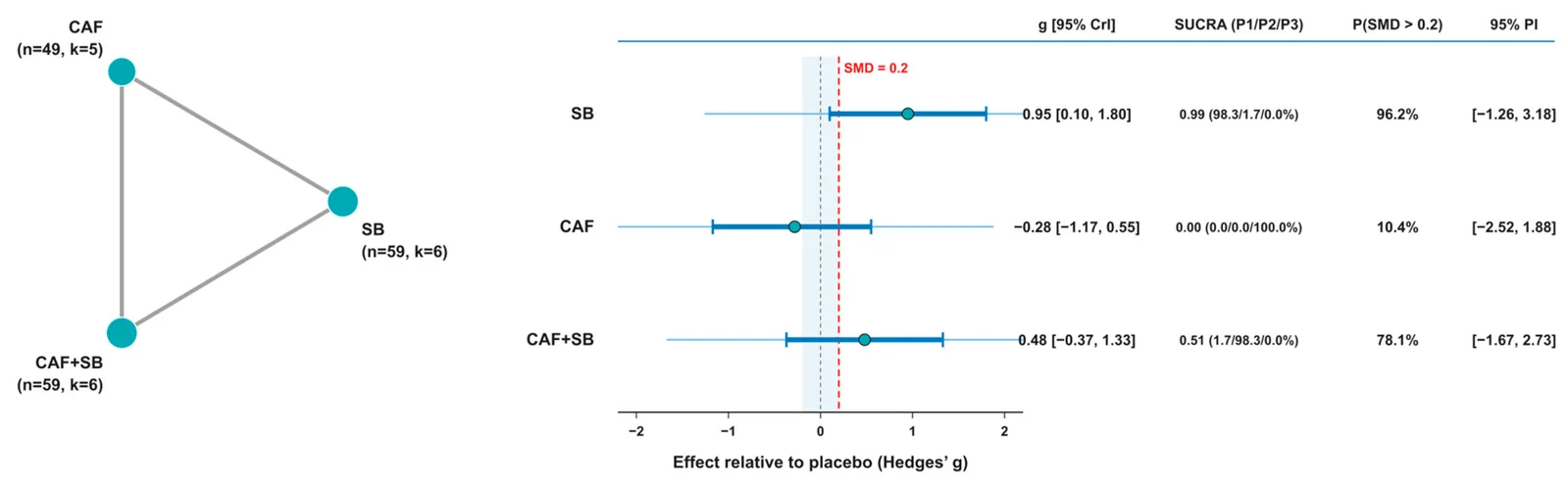
Network geometry and treatment effects on blood pH. Notes: CAF: Caffeine; SB: Sodium bicarbonate; CAF + SB: Caffeine and sodium bicarbonate co-ingestion; CrI: Credible interval; PI: Prediction interval; SUCRA: Surface under the cumulative ranking curve; k: Number of included studies; n: Number of participants. Effects are expressed as standardized mean differences (Hedges’ g) relative to placebo. CAF and SB estimates were derived exclusively from single-supplement arms within eligible co-supplementation trials and do not represent their broader supplement-specific evidence bases. The vertical dashed line indicates no effect (g = 0), and the shaded region represents a predefined small-effect threshold (g = 0.20). P(SMD > 0.20) denotes the posterior probability of exceeding this threshold. SUCRA values reflect the probability of each treatment ranking best, second, or third. Node sizes are proportional to sample size, and edge thickness reflects the number of direct comparisons.

For blood pH, SB produced a credible increase relative to placebo (g = 0.95, 95% CrI [0.10, 1.80]), whereas no clear effects were observed for caffeine (g = −0.28, 95% CrI [−1.17, 0.55]) or co-ingestion (g = 0.48, 95% CrI [−0.37, 1.33]). Pairwise contrasts indicated higher blood pH following SB than following caffeine or co-ingestion. Accordingly, SB ranked highest for increasing blood pH (SUCRA = 0.99) (Figure 5; see also Supplementary Material S4 for the pairwise comparisons plot).

#### 3.4.5. Blood Lactate

**Figure 6:**
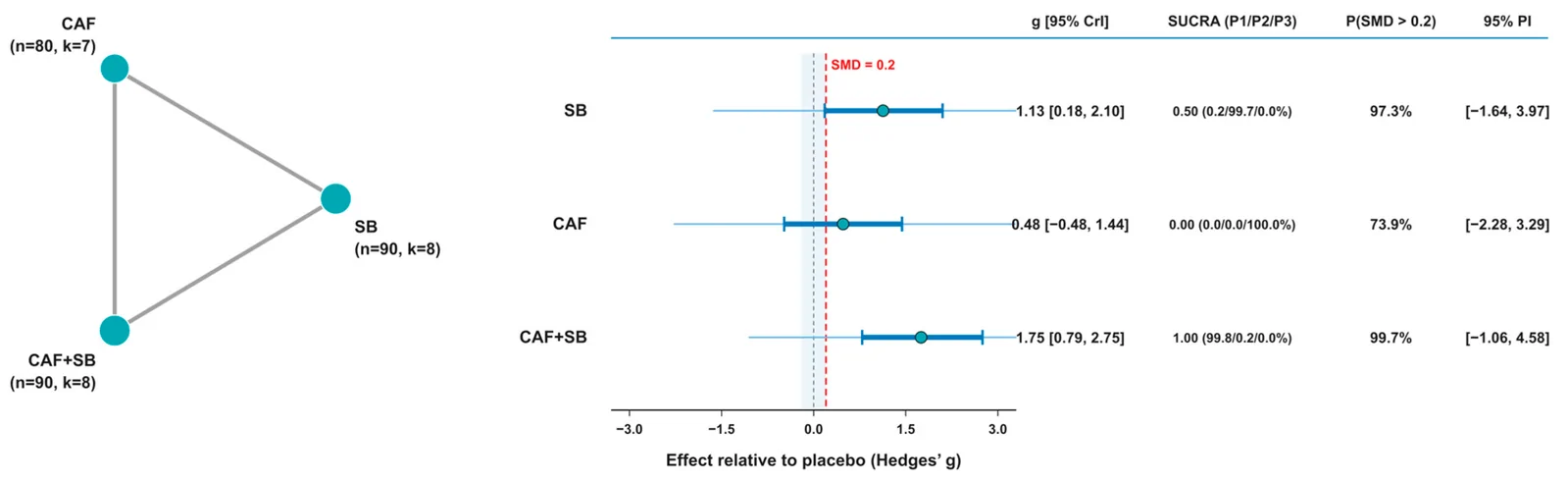
Network geometry and treatment effects on blood lactate. Notes: CAF: Caffeine; SB: Sodium bicarbonate; CAF + SB: Caffeine and sodium bicarbonate co-ingestion; CrI: Credible interval; PI: Prediction interval; SUCRA: Surface under the cumulative ranking curve; k: Number of included studies; n: Number of participants. Effects are expressed as standardized mean differences (Hedges’ g) relative to placebo. CAF and SB estimates were derived exclusively from single-supplement arms within eligible co-supplementation trials and do not represent their broader supplement-specific evidence bases. The vertical dashed line indicates no effect (g = 0), and the shaded region represents a predefined small-effect threshold (g = 0.20). P(SMD > 0.20) denotes the posterior probability of exceeding this threshold. SUCRA values reflect the probability of each treatment ranking best, second, or third. Node sizes are proportional to sample size, and edge thickness reflects the number of direct comparisons.

For blood lactate, SB (g = 1.13, 95% CrI [0.18, 2.10]) and co-ingestion (g = 1.75, 95% CrI [0.79, 2.75]) produced credible increases relative to placebo, whereas no clear effect was observed for caffeine (g = 0.48, 95% CrI [−0.48, 1.44]). Pairwise contrasts indicated higher blood lactate following co-ingestion than following SB alone, with both conditions producing higher values than caffeine. Accordingly, co-ingestion ranked highest for increasing blood lactate (SUCRA = 1.00) (Figure 6; see also Supplementary Material S4 for the pairwise comparisons plot).

### 3.5. Exploratory Subgroup Analyses of Exercise Performance

Exploratory subgroup analyses suggested possible variation in treatment effects according to training status, whereas patterns related to supplement form and exercise type were less clear. Among trained/athlete populations, caffeine (g = 0.24, 95% CrI [0.05, 0.44]) and co-ingestion (g = 0.22, 95% CrI [0.03, 0.42]) produced small credible improvements relative to placebo, whereas SB showed no clear effect (g = 0.03, 95% CrI [−0.16, 0.23]). Among recreationally active individuals, SB produced the largest effect (g = 1.05, 95% CrI [0.64, 1.42]), followed by co-ingestion (g = 0.45, 95% CrI [0.09, 0.80]), while caffeine showed no clear effect (g = 0.04, 95% CrI [−0.33, 0.40]). Subgroup-specific pairwise contrasts favored caffeine and co-ingestion over SB among trained/athlete populations, whereas SB was favored over both caffeine and co-ingestion among recreationally active individuals; co-ingestion was also favored over caffeine in the latter subgroup.

For supplement form, co-ingestion showed a small credible effect when administered in solid form (g = 0.34, 95% CrI [0.06, 0.60]), whereas no clear effects were observed for the other active conditions or for interventions administered in liquid form. Pairwise contrasts among active conditions remained uncertain within both formulation subgroups.

For exercise type, co-ingestion showed a small credible improvement in maximal or explosive neuromuscular performance (g = 0.26, 95% CrI [0.07, 0.48]), while the effects of SB (g = 0.13, 95% CrI [0.03, 0.35]) and caffeine (g = 0.17, 95% CrI [0.04, 0.38]) were credible but below the predefined small-effect threshold. Within high-intensity glycolytic performance, only co-ingestion showed a credible effect (g = 0.36, 95% CrI [0.06, 0.67]); estimates for caffeine and SB remained uncertain. Pairwise contrasts among active conditions were inconclusive within both exercise-type subgroups.

**Figure 7:**
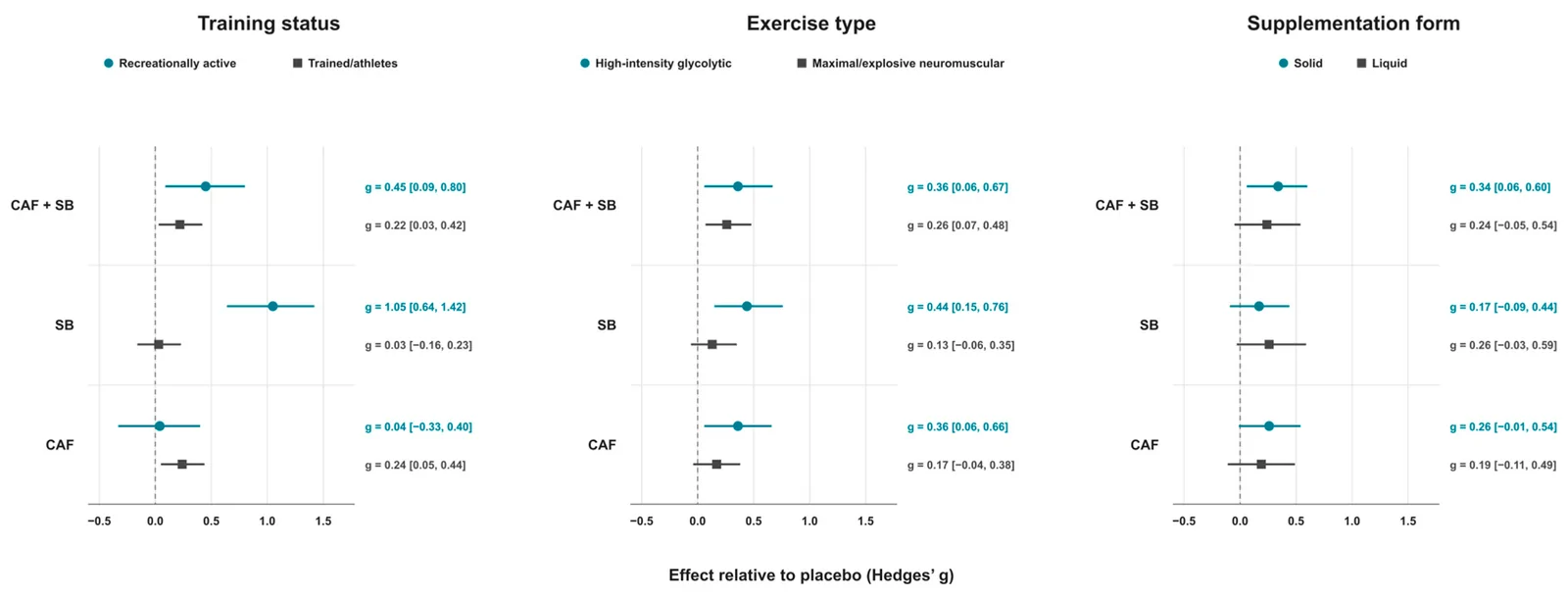
Exploratory subgroup analyses of exercise performance by training status, exercise type, and supplementation form. Notes: CAF, caffeine; SB, sodium bicarbonate; CAF + SB, caffeine and sodium bicarbonate co-ingestion; CrI, credible interval. Points and horizontal lines represent posterior mean effects and 95% CrIs, respectively, expressed as Hedges’ g relative to placebo. The vertical dashed line indicates no effect (g = 0), and colors distinguish subgroup levels. CAF and SB estimates were derived exclusively from single-supplement arms within eligible co-supplementation trials.

Given the limited and unevenly distributed data, these subgroup-specific findings should be considered exploratory and interpreted cautiously. For more details, please refer to Figure 7 and Supplementary Material S4.

### 3.6. Risk of Bias and Methodological Quality

Overall, most studies were judged to be at low risk or with some concerns across domains, with relatively few instances of high risk of bias. The domain of missing outcome data showed consistently low risk across all studies, and outcome measurement was generally considered robust. In contrast, some concerns were frequently identified in the randomization process and selection of the reported results, reflecting limited reporting of allocation procedures and potential selective reporting. Similarly, bias arising from period and carryover effects in crossover trials was often rated as having some concerns. Overall risk of bias was therefore predominantly classified as “some concerns”, with only one study rated at high risk [30] (Supplementary Material S5).

Contour-enhanced funnel plots and Egger’s tests suggested potential small-study effects for several outcomes. For exercise performance, evidence of asymmetry was observed for combined condition and SB, but not caffeine. The S-value for SB was 9.39, suggesting that substantial publication bias would be required to attenuate the observed effect, whereas the S-value was not estimable for the combined condition. For RPE, Egger’s tests were non-significant across all interventions, and S-values were not estimable, consistent with the absence of credible pooled effects. For blood bicarbonate, Egger’s tests did not indicate publication bias. The S-value was not estimable for caffeine despite a significant pooled effect, suggesting a relatively robust finding. In contrast, small S-values for combined condition and SB indicate lower robustness. For blood pH, asymmetry was detected for combined condition and SB, but the main credible effect was observed only for SB; therefore, publication-bias findings for non-credible effects should be interpreted cautiously. For blood lactate, Egger’s tests suggested potential asymmetry across interventions. The S-value was not interpretable for caffeine due to the absence of a credible pooled effect. In contrast, non-estimable S-values for SB and the combined condition, alongside their credible effects, suggest that these findings may be relatively robust to plausible levels of publication bias.

Overall, publication-bias diagnostics suggested some evidence of asymmetry, particularly for performance, blood pH, and blood lactate outcomes. However, interpretation was limited by the small number of studies per treatment and outcome, and S-value results should be interpreted according to whether the corresponding pooled effect was credible. Detailed results are provided in Supplementary Material S6.

The mean modified PEDro score across included studies was 7.6, indicating good methodological quality. For more information, please refer to Supplementary Material S7.

### 3.7. Sensitivity Analyses

Across outcomes, almost all comparisons showed no statistically significant differences between direct and indirect estimates (*p* > 0.05), providing no clear evidence of inconsistency. Notably, the proportion of direct evidence was consistently high (≥0.95 across all comparisons), reflecting the relatively simple network structure and the predominance of direct comparisons. Consequently, indirect estimates were often derived from limited pathways, which in some cases resulted in noticeable numerical discrepancies between direct and indirect estimates, potentially indicating model instability. Furthermore, given the modest number of available studies within each outcome, the statistical power to detect inconsistency may be limited. Therefore, these findings should be interpreted as supportive rather than definitive. Detailed results are provided in Supplementary Material S8.

Sensitivity analyses using alternative within-participant correlations (*r* = 0.20 and 0.80) yielded results broadly consistent with the primary analysis. At *r* = 0.80, the 95% CrI for the effect of co-ingestion on RPE narrowly excluded zero (*g* = −0.36, 95% CrI [−0.72, −0.01]). At *r* = 0.20, the 95% CrI for the effect of caffeine on blood bicarbonate included zero (*g* = −0.75, 95% CrI [−1.59, 0.02]). At the same correlation, the 95% HPD intervals for the caffeine versus co-ingestion contrast in blood bicarbonate and the SB versus co-ingestion contrast in blood pH also included zero. All other estimates were materially unchanged. Overall, the main findings were largely robust to alternative assumptions regarding within-participant correlation, although several estimates were sensitive to the assumed value of *r*. For more details, please refer to Supplementary Material S9.

## 4. Discussion

The present network meta-analysis evaluated caffeine–SB co-ingestion within trials specifically designed to investigate the combined strategy. Using placebo as the common reference, embedded caffeine-only and SB-only arms provided component comparators for assessing the incremental value of co-ingestion under relatively aligned experimental conditions. All three active conditions showed small credible effects relative to placebo within this restricted network, but contrasts among active conditions did not demonstrate that co-ingestion was clearly superior to either component alone. Importantly, the prediction intervals were wide and crossed zero for all three active conditions, indicating considerable uncertainty in the effects that may be expected in future studies and across different exercise contexts. Accordingly, the principal inference is that co-ingestion provides no clear additional ergogenic benefit, while the component-specific estimates should be interpreted as average effects within this limited and heterogeneous trial network and considered alongside broader evidence syntheses for caffeine and SB.

### 4.1. Interaction Between Caffeine and Sodium Bicarbonate: Potentially Complementary but Not Clearly Additive

A key objective of this study was to determine whether co-ingestion provided an additional ergogenic benefit beyond either supplement alone. Despite a strong theoretical rationale, the findings provided no clear evidence that co-ingestion was superior to the individual components. Although the underlying mechanisms were not directly evaluated in the present meta-analysis, the observed metabolic response patterns are compatible with distinct and potentially complementary physiological actions.

Based on established physiological evidence, caffeine primarily influences central nervous system function by reducing perceived exertion and increasing neural drive [1,2], whereas SB enhances extracellular buffering capacity and facilitates hydrogen-ion efflux [3,6]. Although these mechanisms operate through different physiological pathways, improvement in one system may not produce a proportional performance gain when other limiting factors become dominant. Exercise performance reflects the interaction of metabolic, neuromuscular, and perceptual constraints rather than the simple sum of isolated physiological effects [36,37]. Taken together, this framework may help explain why targeting distinct physiological pathways did not consistently yield an additive performance benefit across heterogeneous exercise tasks.

### 4.2. Mechanistic Interpretation: Metabolic Activation and Buffering Balance

SB alone and co-ingestion were associated with numerical increases in blood lactate and pH relative to placebo, although the effect of co-ingestion on blood pH remained uncertain. This pattern is compatible with enhanced extracellular buffering and lactate efflux. In contrast, caffeine reduced blood bicarbonate, possibly reflecting increased metabolic turnover and greater reliance on glycolytic energy pathways [2].

Notably, co-ingestion resulted in the largest increase in blood lactate, which may reflect the combined influence of caffeine-enhanced glycolytic flux and metabolic activation with SB-mediated buffering of hydrogen ions and facilitation of lactate efflux. This interpretation aligns with previous evidence showing that metabolic alkalosis can increase lactate production and efflux during high-intensity exercise [3,38]. At the same time, co-ingestion produced a numerically intermediate increase in blood pH rather than the largest increase, which may be compatible with a dynamic balance between acid production and extracellular buffering. Co-ingestion may therefore support acid–base regulation under increased metabolic stress rather than simply maximizing alkalosis.

However, these pronounced physiological changes did not translate into proportionally greater performance gains. This dissociation indicates that greater metabolic perturbation is not inherently ergogenic, and that performance outcomes likely reflect a balance between enhanced energy turnover and multiple fatigue-related constraints.

### 4.3. Training Status as a Potential Moderator

Exploratory subgroup analyses suggested possible variation in supplementation responses according to training status. Among trained individuals, caffeine and co-ingestion showed small but credible effects relative to placebo, whereas SB alone showed no clear effect. In contrast, SB produced the largest estimated effect among recreationally active individuals.

This pattern may partly reflect differences in baseline physiological capacity. Trained athletes generally exhibit greater intrinsic buffering capacity, fatigue resistance, and task-specific pacing experience, which could reduce the relative contribution of exogenous buffering [39,40]. Conversely, recreationally active individuals may have greater scope for improvement in acid–base regulation and could therefore be more responsive to SB. These physiological differences provide a plausible, although not directly tested, explanation for the observed subgroup pattern.

Importantly, this pattern is broadly consistent with a previous SB meta-analysis based on a larger body of evidence [4], which reported greater ergogenic benefits in recreationally active individuals than in trained athletes. Although relatively few studies contributed to the recreationally active subgroup in the present analysis, convergence with this broader evidence base supports the plausibility of the observed pattern. Nevertheless, these findings should be interpreted cautiously and considered exploratory pending further confirmation.

### 4.4. Exercise Type and Performance Specificity

Exploratory subgroup analyses did not reveal a consistent pattern of variation according to exercise type. In maximal/explosive neuromuscular tasks, all three active conditions showed credible improvements relative to placebo, although the estimates for caffeine and SB remained below the predefined small-effect threshold. In contrast, only co-ingestion showed a credible effect in high-intensity glycolytic tasks, while the estimates for caffeine and SB remained uncertain. This uncertainty in the single-supplement estimates is broadly consistent with findings from a recent randomized crossover trial by Williams et al. [41], which reported no clear ergogenic effects of caffeine or SB on repeated knee-extension or chest-press performance in resistance-trained adults. Collectively, these findings may reflect the complexity of exercise performance, which cannot always be cleanly divided into distinct mechanistic categories. Differences in exercise protocols, duration, metabolic demands, and pacing strategies may also have contributed to variation within each exercise domain. Accordingly, within this focused co-supplementation trial network, the overall pooled estimates should be interpreted as average effects across heterogeneous exercise contexts rather than as uniform effects applicable to each task, and the limited evidence precludes firm task-specific conclusions.

### 4.5. Supplement Form, Timing, and Individual Responsiveness

Exploratory analyses provided no clear evidence of differences between solid and liquid supplementation forms. Co-ingestion showed a small credible effect relative to placebo when administered in solid form, but pairwise contrasts among the active conditions remained uncertain within both formulation subgroups. Nevertheless, the observed pattern may partly reflect differences in absorption kinetics and gastrointestinal tolerance, as solid formulations are generally absorbed more gradually and may offer better tolerability, particularly for SB [42]. The available evidence was insufficient to establish a clear advantage of either delivery form.

Supplement timing may also influence the effects of co-ingestion, although it could not be formally examined because of limited and heterogeneous reporting. Caffeine typically reaches peak plasma concentrations within approximately 30–60 min, whereas SB generally requires approximately 60–120 min, or a staggered ingestion protocol, to achieve peak alkalosis [2,3,4]. Misalignment between these time courses may therefore attenuate the potential interaction between the two supplements.

Inter-individual variability in caffeine responsiveness should also be considered. Some individuals may obtain greater performance benefits from caffeine than others, which could influence the additional benefit observed when caffeine is combined with SB. However, the absence of improvement in a single caffeine–placebo comparison may reflect normal day-to-day variation rather than a consistent non-response, and repeated testing suggests that stable non-response may be less common than previously assumed [43]. Responsiveness may nevertheless vary according to dose and timing, habitual caffeine intake, physiological state, and genetic factors related to caffeine metabolism or adenosine signaling [44]. Because the included studies reported group-level data, the present analysis could not determine whether individual caffeine responsiveness influenced the effects of co-ingestion.

Accordingly, staggered ingestion strategies, in which SB is consumed earlier and caffeine closer to exercise onset, may better align their physiological effects. Dividing the SB dose into several smaller administrations may also facilitate more stable alkalosis and reduce gastrointestinal discomfort [3,4]. Finally, variation in the relative doses of caffeine and SB across studies may have contributed to heterogeneous responses. Future studies should therefore optimize dosing and timing while incorporating repeated within-participant assessments to characterize individual responses to caffeine, SB, and their combination.

### 4.6. Strengths and Limitations

This study has several strengths. First, restricting eligibility to co-supplementation trials provided a focused and relatively aligned experimental context for evaluating the incremental effect of co-ingestion relative to either component alone. The Bayesian arm-based network meta-analysis enabled comparisons among the active conditions within a unified framework, with placebo serving as the common reference. Second, the inclusion of both performance and physiological outcomes supported a mechanistic interpretation of the findings. Third, sensitivity analyses yielded largely consistent results across alternative assumptions, supporting the general robustness of the main findings.

However, several limitations should be acknowledged. The focused eligibility criterion restricted the caffeine-only and SB-only estimates to component arms within co-supplementation trials; consequently, these estimates do not represent the complete evidence base for either supplement used independently. Within this evidence base, the number of included studies was modest, particularly for secondary outcomes and exploratory subgroup analyses, limiting the precision of some estimates. Exercise performance was also pooled across protocols with differing metabolic, neuromuscular, and task-specific demands. Although Hedges’ *g* placed these outcomes on a common statistical scale and exploratory analyses examined two exercise domains, their underlying clinical and physiological heterogeneity remained. Moreover, the primary arm-based model did not directly support formal inconsistency and confidence assessments. Although supplementary contrast-based analyses showed no clear evidence of inconsistency, the simple network structure and limited number of studies reduced the informativeness of these assessments and did not permit a sufficiently reliable application of CINeMA. In addition, heterogeneity in supplementation protocols, including timing, dosing, and ingestion strategies, could not be fully explored. Finally, most studies included male or mixed-sex populations, limiting the generalizability of the findings to female athletes.

### 4.7. Practical Implications

From a practical perspective, co-ingestion did not provide a clear performance advantage over either caffeine or SB alone within the eligible co-supplementation trials. Although combined use may be considered in selected high-intensity exercise contexts, the subgroup evidence remains exploratory, and the more pronounced metabolic responses observed with co-ingestion did not translate into proportionally greater performance benefits. Decisions regarding combined supplementation should therefore be individualized according to exercise demands, dosing and timing, prior individual responses, and gastrointestinal tolerance to SB. Recommendations concerning caffeine or SB used independently should continue to be informed by their broader supplement-specific evidence bases.

## 5. Conclusions

Evidence from the eligible co-supplementation trials indicated that caffeine, SB, and their co-ingestion each produced small but credible improvements in exercise performance relative to placebo. However, co-ingestion did not demonstrate a clear ergogenic advantage over either supplement alone. Although the combined strategy elicited more pronounced metabolic responses, these changes did not translate into proportionally greater performance benefits. Future research should clarify how dosing, timing, exercise demands, and individual responsiveness influence the effectiveness and tolerability of combined supplementation.

## Figures and Tables

**Table 1:** Characteristics and outcomes of studies examining combined caffeine and sodium bicarbonate supplementation.

Study; Study Design	Exercise Protocol	Sample; Mean Age (y), Body Weight (kg); Training Status	Supplement Protocol (Dose, Form, Timing)	Performance	RPE	Blood Markers
Carr et al. (2011) [3] RDB Crossover	2000-m rowing TT	n = 8 (6M/2F), trained rowers	CAF 6 mg/kg + SB 0.3 g/kg (capsules; SB: −90 min & CAF: −30 min)	CAF + SB vs. PLA: Time ↔; Power ↔ CAF vs. PLA: Time ↔; Power ↑ SB vs. PLA: Time ↔; Power ↔ Power: CAF > CAF + SB/SB	N/A	CAF + SB vs. PLA: HCO_3_^−^ ↑; pH ↑; Lactate ↑. CAF vs. PLA: HCO_3_^−^ ↔; pH ↔; Lactate ↔. SB vs. PLA: HCO_3_^−^↑; pH ↑; Lactate ↔.
Christensen et al. (2014) [7] RDB Crossover	6-min rowing test	n = 12 (11M/1F), elite rowers	CAF 3 mg/kg + SB 0.3 g/kg (SB: capsules; −75 min & CAF: pills; −45 min	CAF + SB vs. PLA: Distance ↑; Power ↑ CAF vs. PLA: Distance ↑; Power ↑ SB vs. PLA: Distance ↔; Power ↔ Distance: CAF/CAF + SB > SB Power: CAF/CAF + SB > SB	RPE ↔	N/A
Correia-Oliveira et al. (2023) [29] RDB Crossover	4-km cycling TT	n = 10 (N/A), moderately trained cyclists	CAF 5 mg/kg + SB 0.3 g/kg (capsules; SB: −100 min & CAF: −60 min)	CAF + SB vs. PLA: Time ↓; Power ↑ CAF vs. PLA: Time ↔; Power ↔ Performance (Time, mean power output): CAF + SB > CAF	N/A	CAF + SB vs. PLA: HCO_3_^−^ ↑; pH ↑; Lactate ↔. SB vs. PLA: HCO_3_^−^ ↑; pH ↑; Lactate ↔.
Felippe et al. (2016) [5] RDB Crossover	3 special judo fitness tests	n = 10 (M), judo athletes	CAF 6 mg/kg + SB 0.3 g/kg (capsules; SB: −120, −90, −60 min & CAF: −60 min)	CAF + SB vs. PLA: Throws ↑ CAF vs. PLA: Throws ↔ SB vs. PLA: Throws ↑	All vs. PLA: RPE ↔	All vs. PLA: Lactate ↑
Ferragut et al. (2024) [8] RDB Crossover	4 × 30-s Wingate	n = 25 (13M/12F), recreationally trained	CAF 3 mg/kg + SB 0.3 g/kg (solution; SB: −120, −90 min & CAF: −60 min)	CAF + SB vs. PLA: Peak power ↑; Mean power ↔ CAF vs. PLA: Peak power ↑; Mean power ↔ SB vs. PLA: Peak power ↑; Mean power ↑	N/A	CAF + SB vs. PLA: Lactate ↑. CAF vs. PLA: Lactate ↔. SB vs. PLA: Lactate ↑. Lactate: CAF + SB > CAF; SB > CAF.
Higgins et al. (2016) [32] RDB Crossover	Cycling TTE	n = 13 (M), recreationally active	CAF 5 mg/kg + SB 0.3 g/kg (solution; −60 min)	All vs. PLA: TTE ↔	All vs. PLA: RPE ↔	CAF + SB vs. PLA: Lactate ↑; HCO_3_^−^ ↑; pH ↑. CAF vs. PLA: Lactate ↔; HCO_3_^−^ ↔; pH ↔. SB vs. PLA: Lactate ↑; HCO_3_^−^ ↑; pH ↑.
Kaçoğlu et al. (2024) [33] RDB Crossover	Isometric mid-thigh pull test	n = 19 (M), recreationally active	CAF 6 mg/kg + SB 0.3 g/kg (solution; −60 min)	CAF + SB vs. PLA: Peak force: ↔; Relative force: ↔ CAF vs. PLA: Peak force: ↔; Relative force: ↑ SB vs. PLA: Peak force: ↔; Relative force: ↔ Relative force: CAF > SB/CAF + SB	N/A	N/A
Kilding et al. (2012) [9] RDB Crossover	3-km cycling TT	n = 10 (M), trained cyclists	CAF 3 mg/kg + SB 0.3 g/kg (capsules; SB: −120, −90 min & CAF: −60 min)	All vs. PLA: Time ↓; Power ↑	All vs. PLA: RPE ↔	CAF + SB vs. PLA: HCO_3_^−^ ↑; pH ↑; Lactate ↔. CAF vs. PLA: HCO_3_^−^ ↔; pH ↔; Lactate ↔. SB vs. PLA: HCO_3_^−^ ↑; pH ↑; Lactate ↔.
Moesgaard et al. (2024) [34] RDB Crossover	15-s cycling sprint + 6-min TT	n = 12 (6M/6F), endurance-trained	CAF 3 mg/kg + SB 0.3 g/kg (beverage; −60 min)	CAF + SB vs. PLA: Sprint mean power ↔; Sprint peak power ↔; TT mean power ↑; MVC ↔ CAF vs. PLA: Sprint mean power ↔; Sprint peak power ↑; TT mean power ↑ MVC ↔ SB vs. PLA: Sprint mean power ↔; Sprint peak power ↔; TT mean power ↔; MVC ↔	N/A	CAF + SB vs. PLA: HCO_3_^−^ ↑; pH ↑. CAF vs. PLA: HCO_3_^−^ ↓; pH ↓. SB vs. PLA: HCO_3_^−^ ↑; pH ↑
Montalvo-Alonso et al. (2024) [35] RDB Crossover	65% and 85% 1-RM bench press and back squat to failure	n = 28 (14M/14F), resistance-trained	CAF 3 mg/kg + SB 0.3 g/kg (solution; SB: −120, −90 min & CAF: −60 min)	CAF + SB vs. PLA: Repetitions ↔ CAF vs. PLA: Repetitions ↑ SB vs. PLA: Repetitions ↔	N/A	N/A
Pruscino et al. (2008) [30] RDB Crossover	2 × 200-m freestyle TT	n = 6 (M), elite swimmers	CAF 6 mg/kg + SB 0.3 g/kg (capsules; SB: −120 first time, other 6 doses over 90 min & CAF: −45 min)	All vs. PLA: Time ↔	N/A	All vs. PLA: HCO_3_^−^ ↓; pH ↓; Lactate ↓. HCO_3_^−^: TT1: Decline of CAF + SB > CAF/SB; TT2: Decline of SB > CAF/CAF + SB. pH: TT1: Decline of CAF + SB > SB; Decline of CAF > SB.
Rezaei et al. (2019) [6] RDB Crossover	Karate-specific aerobic test to exhaustion	n = 8 (N/A), competitive karatekas	CAF 6 mg/kg + SB 0.3 g/kg (capsules; SB: 0.3 g/kg/day × 3 + 0.1 g/kg −120, −90, −60 min & CAF: −50 min)	All vs. PLA: TTE ↑	All vs. PLA: RPE ↔	CAF + SB vs. PLA: Lactate ↔. CAF vs. PLA: Lactate ↔. SB vs. PLA: Lactate ↔.
Ziyaiyan et al. (2023) [31] RDB Crossover	Cindy CrossFit workout	n = 20 (M), well-trained	CAF 6 mg/kg + SB 0.3 g/kg (capsules; SB: 0.3 g/kg/day × 3 + 0.1 g/kg −120, −90, −60 min & CAF: −50 min)	All vs. PLA: Repetitions ↔; Strength ↔; Jump ↔	CAF + SB vs. PLA: RPE ↓ CAF vs. PLA: RPE ↔ SB vs. PLA: RPE ↔	N/A

Notes: CAF = caffeine; F = female; g/kg = gram/kilogram; RPE = rating of perceived exertion; RDB = randomized double-blind trial; RSB = randomized single-blind trial; SB = sodium bicarbonate; M = male; min = minute; TT = time trial; TTE = time to exhaustion; IMTP = isometric mid-thigh pull; HCO_3_^−^ = blood bicarbonate concentration; pH = blood pH; lactate = blood lactate concentration; N/A = not applicable/not reported; y = years old; ↑ = significant increase; ↓ = significant decrease; ↔ = no significant change.

## Data Availability

All data analyzed in this study were obtained from previously published studies, which are cited in the manuscript. No new data were generated for this study. The extracted data supporting the findings are available from the corresponding author upon reasonable request.

## Supplementary Materials

The following supporting information can be downloaded at: https://www.mdpi.com/article/10.3390/metabo16090658/s1, Supplementary Material S1: PRISMA 2020 checklist; Supplementary Material S2: Summary of effect size calculation procedures; Supplementary Material S3: Side effects summary; Supplementary Material S4: Differences between groups; Supplementary Material S5: RoB 2 assessment; Supplementary Material S6: Funnel plots; Supplementary Material S7: PEDro assessment; Supplementary Material S8: Incoherence assessment; Supplementary Material S9: Sensitivity analysis for primary results.
