# Supplementary material for: Individual and Combined Effects of Acute Caffeine and Sodium Bicarbonate Supplementation on Exercise Performance and Metabolic Responses: A Bayesian Network Meta-Analysis of Co-Supplementation Trials
Source: Metabolites. 2026 Sep 8;16(9):658. doi: 10.3390/metabo16090658 (PMC13609103; doi:10.3390/metabo16090658)
Supplement: Supplementary file 1 [file metabolites-16-00658-s001.zip › metabolites-4519558-supplementary.pdf]

Electronic Supplementary Material Contents

Number	Material	Page
1	Electronic Supplementary Material S1 (PRISMA 2020 checklist)	P2-5
2	Electronic Supplementary Material S2 (Table. Summary of effect size calculation procedures)	P6-7
3	Electronic Supplementary Material S3 (Side Effects Summary)	P8-9
4	Electronic Supplementary Material S4 (Difference between groups)	P10-12
5	Electronic Supplementary Material S5 (ROB2 Assessment Tool for Risk of Bias)	P13
6	Electronic Supplementary Material S6 (Funnel Plot)	P14-15
7	Electronic Supplementary Material S7 (PEDro Assessment)	P16
8	Electronic Supplementary Material S8 (Incoherence assessment)	P17-18
9	Electronic Supplementary Material S9 (Sensitivity Analysis for Primary results)	P19

Electronic Supplementary Material S1 (PRISMA 2020 checklist)

Section and Topic	Item #	Checklist item	Location where item is reported (on page)
**TITLE: Individual and combined effects of acute caffeine and sodium bicarbonate supplementation on exercise performance and metabolic responses: A Bayesian network meta-analysis of co-supplementation trials**			
Title	1	Identify the report as a systematic review.	P1
**ABSTRACT**			
Abstract	2	See the PRISMA 2020 for Abstracts checklist.	P1-2
**INTRODUCTION**			
Rationale	3	Describe the rationale for the review in the context of existing knowledge.	P2-3
Objectives	4	Provide an explicit statement of the objective(s) or question(s) the review addresses.	P2-3
**METHODS**			
Eligibility criteria	5	Specify the inclusion and exclusion criteria for the review and how studies were grouped for the syntheses.	P3-4
Information sources	6	Specify all databases, registers, websites, organisations, reference lists and other sources searched or consulted to identify studies. Specify the date when each source was last searched or consulted.	P4
Search strategy	7	Present the full search strategies for all databases, registers and websites, including any filters and limits used.	P4
Selection process	8	Specify the methods used to decide whether a study met the inclusion criteria of the review, including how many reviewers screened each record and each report retrieved, whether they worked independently, and if applicable, details of automation tools used in the process.	P3-4
Data collection process	9	Specify the methods used to collect data from reports, including how many reviewers collected data from each report, whether they worked independently, any processes for obtaining or confirming data from study investigators, and if applicable, details of automation tools used in the process.	P3-4
Data items	10a	List and define all outcomes for which data were sought. Specify whether all results that were compatible with each outcome domain in each study were sought (e.g. for all measures, time points, analyses), and if not, the methods used to decide which results to collect.	P4
	10b	List and define all other variables for which data were sought (e.g. participant and intervention characteristics, funding sources). Describe any	P4

Section and Topic	Item #	Checklist item	Location where item is reported (on page)
		assumptions made about any missing or unclear information.	
Study risk of bias assessment	11	Specify the methods used to assess risk of bias in the included studies, including details of the tool(s) used, how many reviewers assessed each study and whether they worked independently, and if applicable, details of automation tools used in the process.	P4-5
Effect measures	12	Specify for each outcome the effect measure(s) (e.g. risk ratio, mean difference) used in the synthesis or presentation of results.	P5
Synthesis methods	13a	Describe the processes used to decide which studies were eligible for each synthesis (e.g. tabulating the study intervention characteristics and comparing against the planned groups for each synthesis (item #5)).	P5-6
	13b	Describe any methods required to prepare the data for presentation or synthesis, such as handling of missing summary statistics, or data conversions.	P5-6
	13c	Describe any methods used to tabulate or visually display results of individual studies and syntheses.	P5-6
	13d	Describe any methods used to synthesize results and provide a rationale for the choice(s). If meta-analysis was performed, describe the model(s), method(s) to identify the presence and extent of statistical heterogeneity, and software package(s) used.	P5-6
	13e	Describe any methods used to explore possible causes of heterogeneity among study results (e.g. subgroup analysis, meta-regression).	P6-7
	13f	Describe any sensitivity analyses conducted to assess robustness of the synthesized results.	P6-7
Reporting bias assessment	14	Describe any methods used to assess risk of bias due to missing results in a synthesis (arising from reporting biases).	P6-7
Certainty assessment	15	Describe any methods used to assess certainty (or confidence) in the body of evidence for an outcome.	P6-7
**RESULTS**			
Study selection	16a	Describe the results of the search and selection process, from the number of records identified in the search to the number of studies included in the review, ideally using a flow diagram.	P7
	16b	Cite studies that might appear to meet the inclusion criteria, but which were excluded, and explain why they were excluded.	P7
Study characteristics	17	Cite each included study and present its characteristics.	P7-12

Section and Topic	Item #	Checklist item	Location where item is reported (on page)
Risk of bias in studies	18	Present assessments of risk of bias for each included study.	P16-17
Results of individual studies	19	For all outcomes, present, for each study: (a) summary statistics for each group (where appropriate) and (b) an effect estimate and its precision (e.g. confidence/credible interval), ideally using structured tables or plots.	P12-16
Results of syntheses	20a	For each synthesis, briefly summarise the characteristics and risk of bias among contributing studies.	P16-17
	20b	Present results of all statistical syntheses conducted. If meta-analysis was done, present for each the summary estimate and its precision (e.g. confidence/credible interval) and measures of statistical heterogeneity. If comparing groups, describe the direction of the effect.	P12-16
	20c	Present results of all investigations of possible causes of heterogeneity among study results.	P15-17
	20d	Present results of all sensitivity analyses conducted to assess the robustness of the synthesized results.	P17
Reporting biases	21	Present assessments of risk of bias due to missing results (arising from reporting biases) for each synthesis assessed.	P16-17
Certainty of evidence	22	Present assessments of certainty (or confidence) in the body of evidence for each outcome assessed.	P17
**DISCUSSION**			
Discussion	23a	Provide a general interpretation of the results in the context of other evidence.	P17-20
	23b	Discuss any limitations of the evidence included in the review.	P20
	23c	Discuss any limitations of the review processes used.	P20
	23d	Discuss implications of the results for practice, policy, and future research.	P20
**OTHER INFORMATION**			
Registration and protocol	24a	Provide registration information for the review, including register name and registration number, or state that the review was not registered.	P3
	24b	Indicate where the review protocol can be accessed, or state that a protocol was not prepared.	P3
	24c	Describe and explain any amendments to information provided at registration or in the protocol.	N/A
Support	25	Describe sources of financial or non-financial support for the review, and the role of the funders or sponsors in the review.	P21

Section and Topic	Item #	Checklist item	Location where item is reported (on page)
Competing interests	26	Declare any competing interests of review authors.	P21
Availability of data, code and other materials	27	Report which of the following are publicly available and where they can be found: template data collection forms; data extracted from included studies; data used for all analyses; analytic code; any other materials used in the review.	N/A

Electronic Supplementary Material S2 (Table. Summary of effect size calculation procedures)

Step	Data condition	Calculation Formula	Description
①	Pre–post data available	$\Delta\mathrm{EXP} = \mathrm{M_{EXPpost}} - \mathrm{M_{EXPpre}}$<br>$\Delta\mathrm{PLA} = \mathrm{M_{PLApost}} - \mathrm{M_{PLApre}}$<br>$\mathrm{MD} = \Delta\mathrm{EXP} - \Delta\mathrm{PLA}$	Calculate the mean change within each condition (experimental: EXP and placebo) and use their difference (MD) as the comparison metric.
②	Only post-intervention data available	$\mathrm{MD} = \mathrm{M_{EXP}} - \mathrm{M_{PLA}}$	When only post values are reported, the mean difference between conditions is used directly.
③	SD of change (for pre–post data)	$\mathrm{SD_{change}} = \sqrt{\mathrm{SD^2{}_{pre}} + \mathrm{SD^2{}_{post}} - \left(2 \times r \times \mathrm{SD_{pre}} \times \mathrm{SD_{post}}\right)}$	Computes the within-subject SD of change using the correlation (r) between pre- and post-measurements.
④	Pooled SD for (*i*) crossover and (*ii*) parallel design	$\mathrm{SD_{pooled}} = \sqrt{\frac{\mathrm{SD^2{}_{EXP}} + \mathrm{SD^2{}_{PLA}}}{2}}$ *(i)*<br>$\mathrm{SD_{pooled}} = \sqrt{\frac{(n_{\mathrm{EXP}}-1)\times SD^2_{\mathrm{EXP}} + (n_{\mathrm{PLA}}-1)\times SD^2_{\mathrm{PLA}}}{n_{\mathrm{EXP}}+n_{\mathrm{PLA}}-2}}$ *(ii)*	Estimates the pooled variability across both conditions.
⑤		$\text{Hedge's g} = \frac{\mathrm{M_{EXP}} - \mathrm{M_{PLA}}}{\mathrm{SD_{pooled}}} \times \left(1 - \frac{3}{4(N-1)-1}\right)$ *(i)*	Standardized mean difference corrected for small-sample bias.

	Effect size (Hedges' *g*) for (*i*) crossover and (*ii*) parallel design	$\text{Hedge's g} = \frac{M_{EXP} - M_{PLA}}{SD_{pooled}} \times \left(1 - \frac{3}{4(n_{EXP}+n_{PLA}-2)-1}\right)$ (*ii*)	
⑥	Standard error (SE) of *g* for (*i*) crossover and (*ii*) parallel design	$SE = \sqrt{\frac{1}{N} + \frac{g^2}{2N}} \times \sqrt{2(1-r)}$ (i)<br>$SE = \sqrt{\frac{n_{EXP}+n_{PLA}}{n_{EXP} \times n_{PLA}} + \frac{g^2}{2(n_{EXP}+n_{PLA})}}$ (*ii*)	(*i*) Adjusts SE to account for within-subject correlation between EXP and placebo conditions. (*ii*) Calculates SE from independent EXP and placebo group sizes, with the harmonic mean accounting for potential imbalance.
⑦	Assumed correlation (*r*)	*r* = 0.50 (primary analysis)<br>*r* = 0.20 and 0.80 (sensitivity)	The same r value was used for both pre-post and between-condition comparisons to ensure consistency and comparability.

Electronic Supplementary Material S3 (Side Effects Summary)

Study	Formulation	SB dosing strategy	Reported	Assessment method	Adverse effects	Main findings
Carr 2011	Capsules	Single bolus (before CAF)	Yes	GI symptoms	GI symptoms present	All subjects reported GI symptoms (e.g., nausea, diarrhea), potentially affecting performance
Christensen 2014	Pills + capsules	Single bolus (before CAF)	Yes	Stomach discomfort	No significant difference	No significant differences in stomach discomfort between conditions
Correia-Oliveira 2023	Capsules	Single bolus (before CAF)	Yes	Questionnaire	Minimal	One participant reported moderate heartburn; no severe symptoms
Felippe 2016	Capsules	Split (−120/−90/−60 min)	Yes	GI questionnaire	GI symptoms present	Some participants reported flatulence and urge to defecate (moderate–high scores)
Ferragut 2024	Liquid (dissolved beverage)	Split (−120/−90 min)	Yes	Side-effects questionnaire	GI symptoms present	$NaHCO_3$ (±CAF) increased GI discomfort
Higgins 2016	Solution	Single bolus	Yes	Abdominal discomfort / gut fullness	GI symptoms present	Higher abdominal discomfort in bicarbonate conditions vs placebo

Kaçoğlu 2024	Solution (dissolved in water)	Single bolus	No	—	Not reported	No adverse-effect data reported
Kilding 2012	Capsules	Split (−120/−90 min)	Yes	Gastric discomfort	Minimal	Low gastric discomfort scores with no meaningful differences
Moesgaard 2024	Beverage	Single bolus	Yes	Side-effect reporting	GI symptoms present	Multiple participants reported GI symptoms (e.g., nausea, diarrhea)
Montalvo-Alonso 2024	Liquid (dissolved beverage)	Split (−120/−90 min)	Yes	Questionnaire	GI symptoms present	$NaHCO_3$ (±CAF) increased GI discomfort
Pruscino 2008	Capsules	Split (multiple doses)	Yes	Self-report	GI symptoms present	Some participants reported stomach cramps, diarrhea, dizziness
Rezaei 2019	Capsules	Loading + split dosing	Yes	GI questionnaire	None	No GI discomfort reported
Ziyaiyan 2023	Capsules	Loading + split dosing	Yes	GI symptom score	GI symptoms present	$NaHCO_3$ conditions increased GI symptoms, but mild and transient

Electronic Supplementary Material S4 (Difference between groups)

Exercise performance:

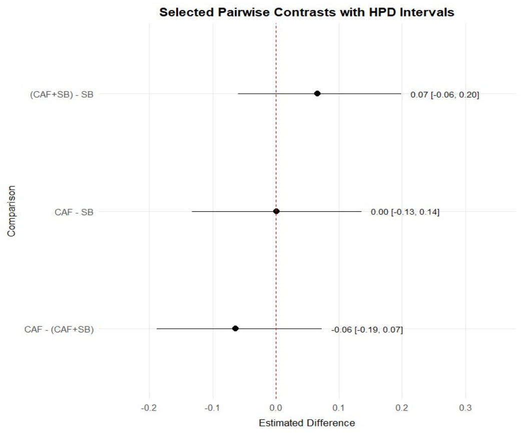

Rating of perceived exertion:

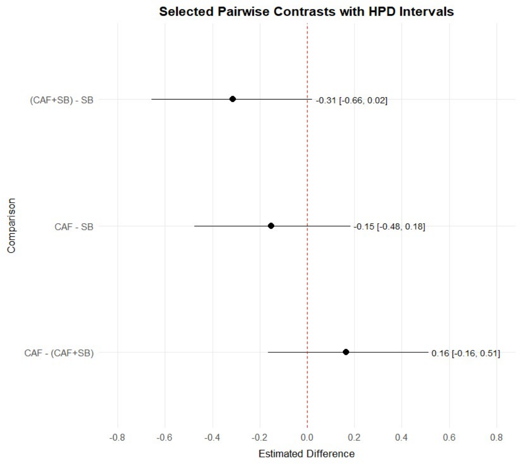

Blood bicarbonate:

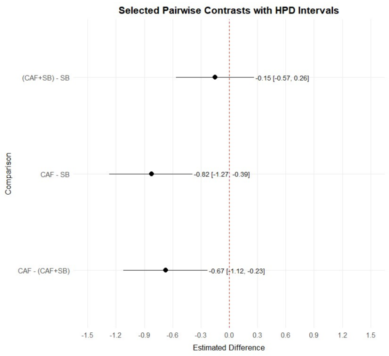

Blood pH:

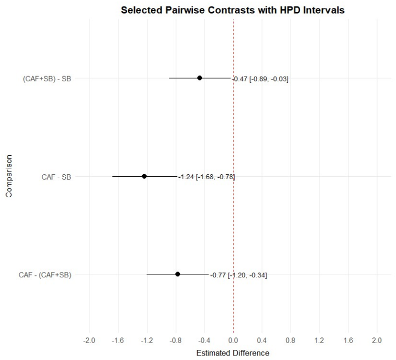

Blood lactate:

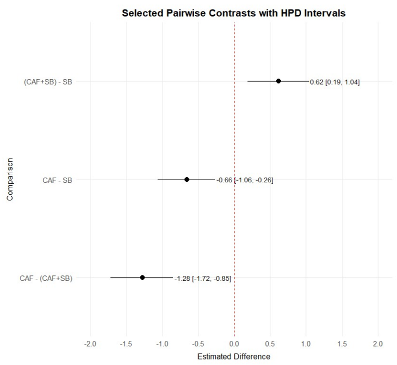

Subgroup differences in exercise performance:

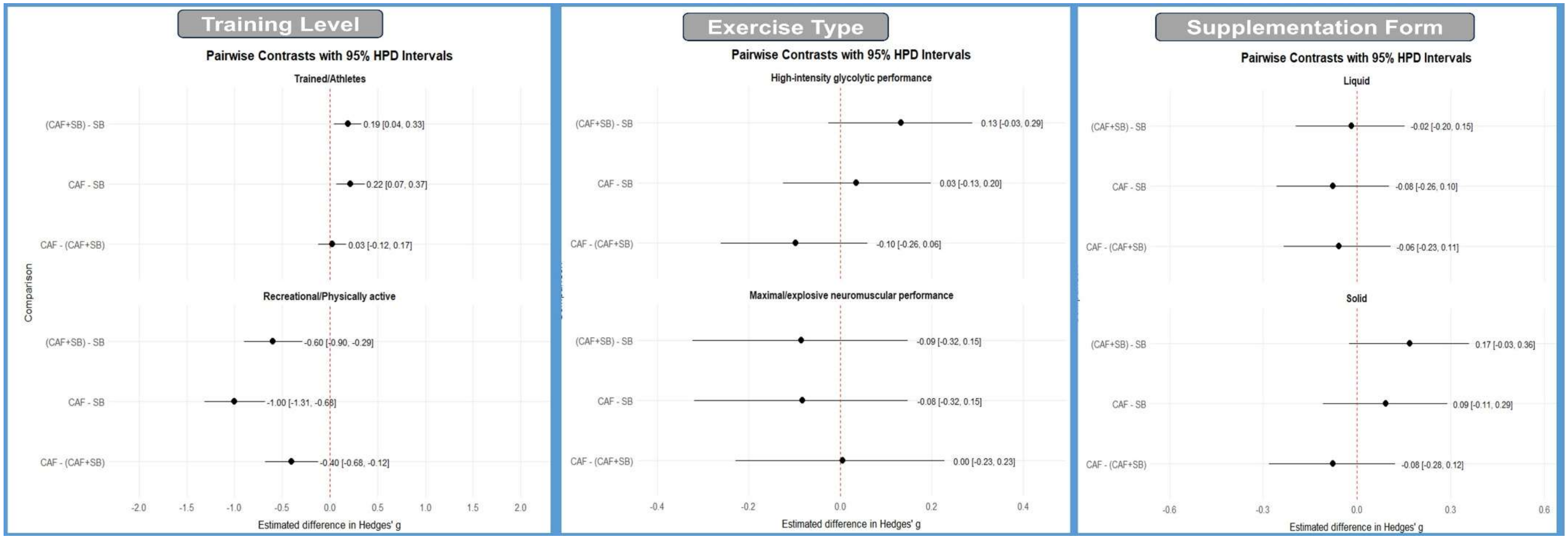

Notes: Hedge's g, the effect size indicators used in the pooled; ***95%CI***, 95% confidence interval; ***The size of each black square***, represents the relative weight of the study in the meta-analysis; ***A***, for exercise performance; ***B***, for core body temperature; ***C*** for thermal sensation; ***D*** for thermal comfort; ***E*** for rating of perceived exertion.

Electronic Supplementary Material S5 (ROB2 Assessment Tool for Risk of Bias)

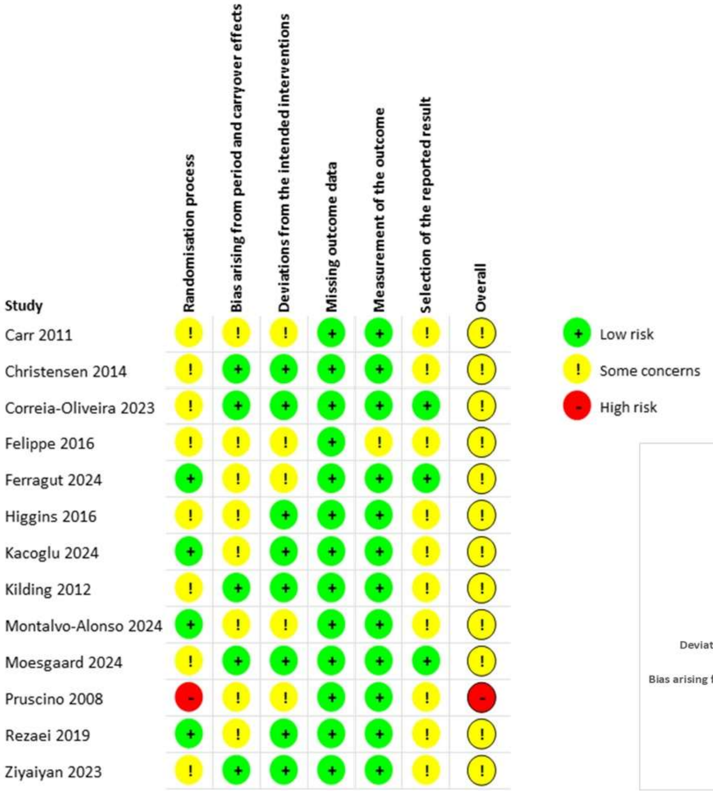

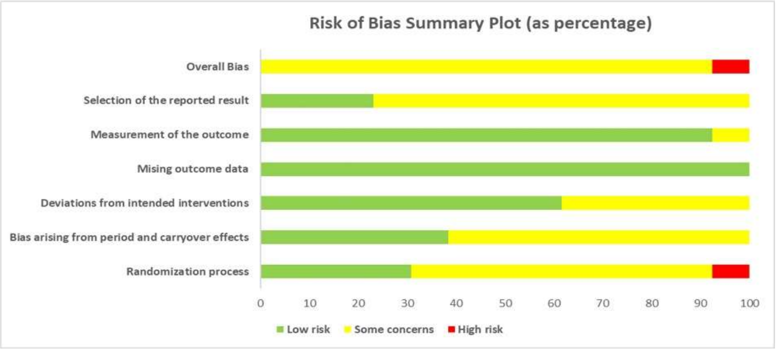

.

Electronic Supplementary Material S6 (Funnel Plot)

Exercise performance:

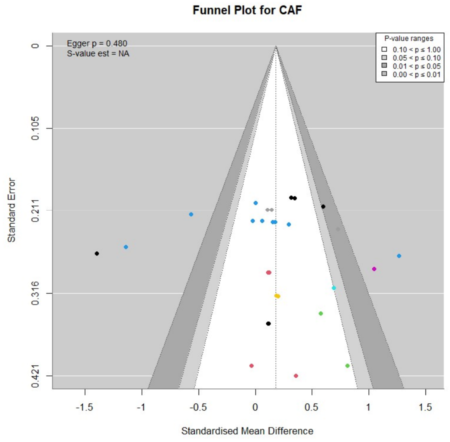

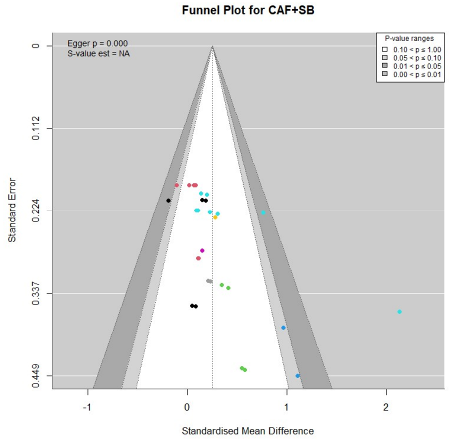

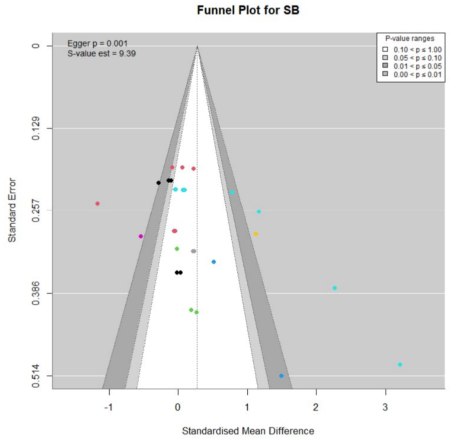

Rating of perceived exertion:

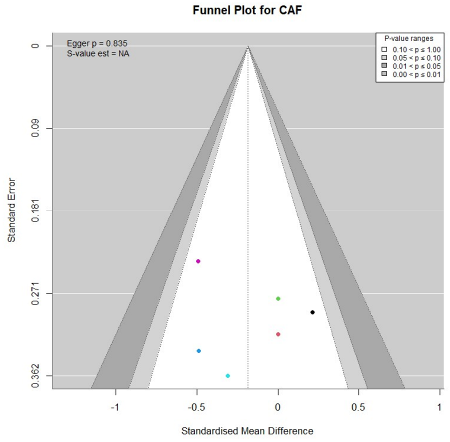

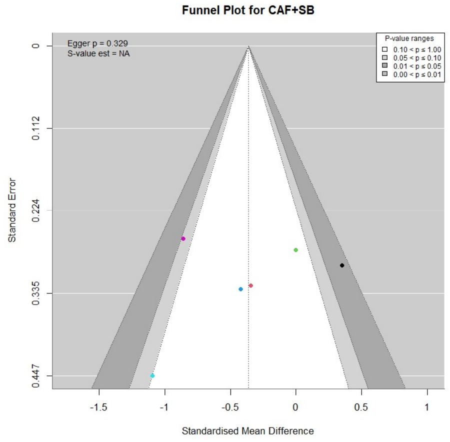

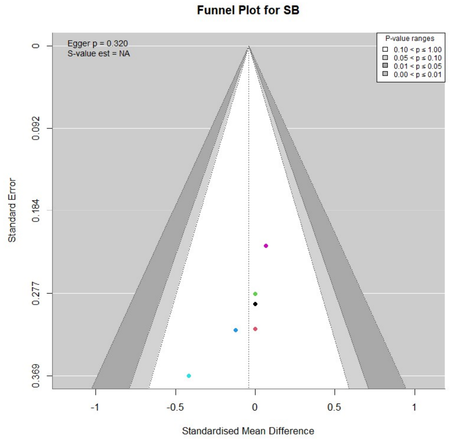

Blood bicarbonate:

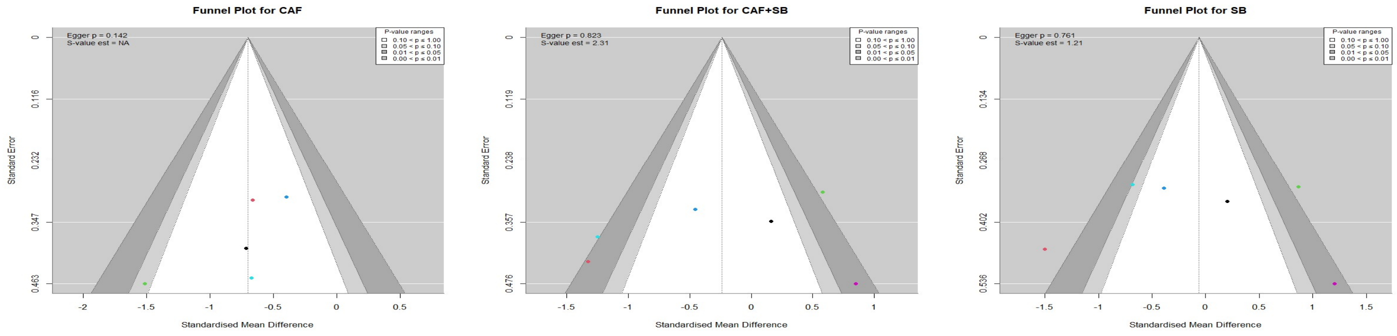

Blood PH:

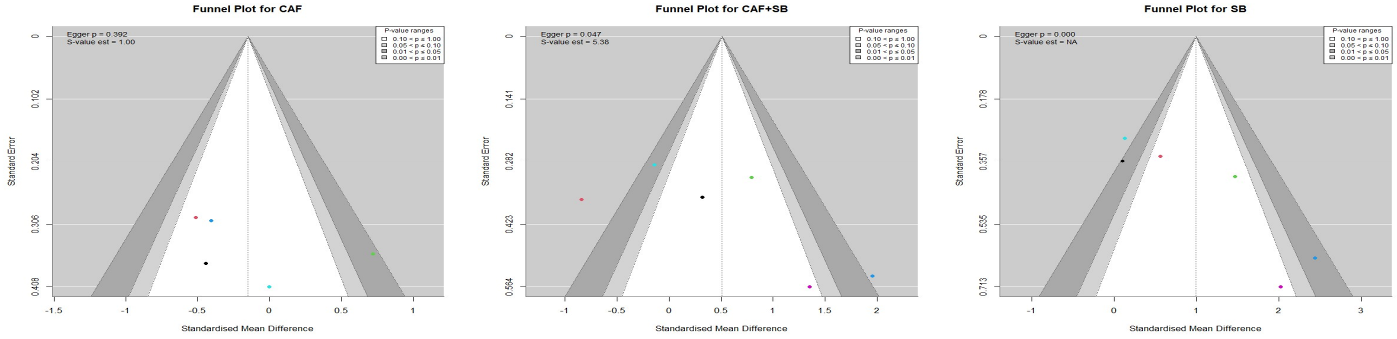

Blood lactate:

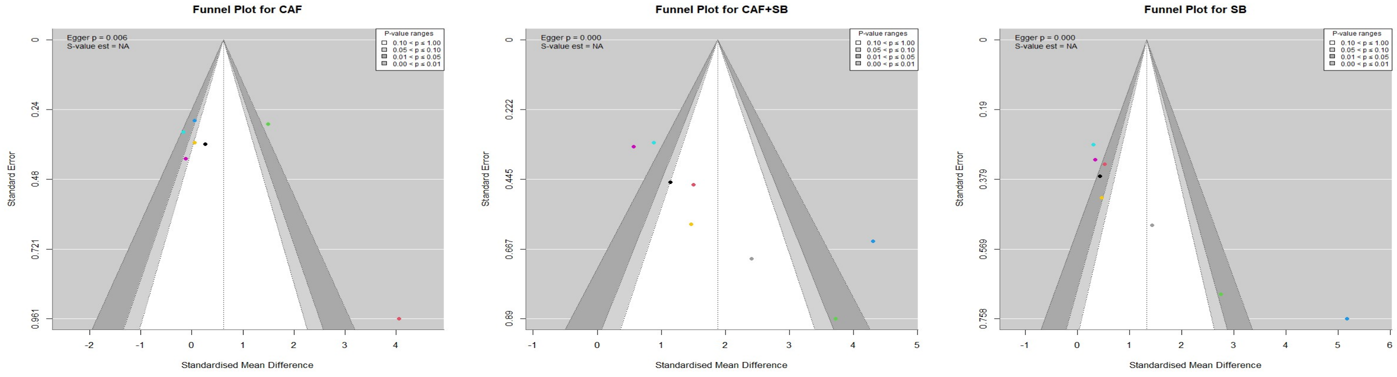

Notes: Colorful circles, represent different effect size points. ***CAF***: caffeine; ***SB***: Sodium bicarbonate.

Electronic Supplementary Material 7 (PEDro Assessment)

Table A. PEDro assessment results of exercise performance researches

Study	Item 1	Item 2	Item 3	Item 4	Item 5	Item 6	Item 7	Item 8	Item 9	Item 10	Item 11	Item 12	Total
Carr 2011	Yes	No	No	No	No	Yes	Yes	Yes	Yes	Yes	Yes	Yes	7
Christensen 2014	Yes	Yes	No	Yes	Yes	No	Yes	Yes	Yes	Yes	Yes	No	8
Correia-Oliveira 2023	Yes	No	No	Yes	Yes	Yes	Yes	Yes	Yes	Yes	Yes	Yes	9
Felippe 2016	Yes	Yes	No	Yes	Yes	Yes	No	Yes	Yes	Yes	Yes	No	8
Ferragut 2024	Yes	Yes	No	Yes	Yes	Yes	Yes	Yes	Yes	Yes	Yes	No	9
Higgins 2016	Yes	No	No	Yes	Yes	No	No	Yes	Yes	Yes	Yes	No	6
Kacoglu 2024	Yes	Yes	Yes	Yes	No	Yes	Yes	Yes	Yes	Yes	Yes	No	9
Kilding 2012	Yes	Yes	No	Yes	Yes	No	No	Yes	Yes	Yes	Yes	No	7
Montalvo-Alonso 2024	Yes	Yes	Yes	No	No	No	Yes	Yes	Yes	Yes	Yes	Yes	8
Moesgaard 2024	Yes	Yes	No	Yes	No	No	Yes	Yes	Yes	Yes	Yes	No	7
Pruscino 2008	Yes	Yes	No	No	No	No	Yes	Yes	Yes	Yes	Yes	No	6
Rezaei 2019	Yes	Yes	No	Yes	No	Yes	Yes	No	Yes	Yes	Yes	No	7
Ziyaiyan 2023	Yes	Yes	Yes	No	Yes	No	Yes	Yes	Yes	Yes	Yes	No	8

Electronic Supplementary Material S8 (Incoherence assessment)

Comparisons	n	Proportion	Estimate	Direct value	Indirect value	Difference	p-value	Pooled p-value
**Exercise**								0.4248
**CAF+SB:CAF**	12	0.98	-0.0669	-0.0790	0.6303	-0.7093	0.4705	
**SB:CAF**	12	0.98	-0.0962	-0.0813	-0.8978	0.8165	0.3967	
**CAF+SB:SB**	13	1	0.0292	0.0278	1.4063	-1.3786	0.7166	
**RPE**								N/A`
**CAF+SB:CAF**	6	1	-0.1737	-0.1813	4.6488	-4.8301	0.2730	
**SB:CAF**	6	1	0.1521	0.1503	N/A	N/A	N/A	
**CAF+SB:SB**	6	1	-0.3257	-0.3268	0.3058	-0.6326	0.8807	
**Blood bicarbonate**								0.6577
**CAF+SB:CAF**	5	0.96	0.7479	0.7266	1.3025	-0.5759	0.7395	
**SB:CAF**	5	0.96	0.9203	0.9539	0.0411	0.9128	0.5996	
**CAF+SB:SB**	6	1	-0.1724	--0.1962	28.5282	-28.7244	0.0069	
**Blood PH**								0.0638
**CAF+SB:CAF**	5	0.95	0.8384	0.9163	-0.6041	1.5204	0.2359	
**SB:CAF**	5	0.93	1.3417	1.2102	3.0818	-1.8717	0.1032	
**CAF+SB:SB**	6	1	-0.5034	-0.5299	12.5718	-13.1018	0.0345	
**B[la]**								0.6577
**CAF+SB:CAF**	7	0.95	1.3800	1.2919	3.0073	-1.7154	0.2541	
**SB:CAF**	7	0.96	0.7585	0.7533	0.8704	-0.1172	0.9399	
**CAF+SB:SB**	8	1	0.6215	0.5916	9.4172	-8.8256	0.1118	

Notes: B[la]: Blood lactate; ***CAF***: Caffeine; ***CAF+SB*** Caffeine and sodium bicarbonate co-supplementation; ***HR***: Heart rate; ***RPE***: Rating of perceived

exertion; ***TAU***: Sodium bicarbonate; ***N/A***: Not applicable; ***n***: Number of included participants; ***Proportion*****:** Weighting of direct evidence in network estimation (1.00 indicates that all information comes from direct evidence); ***p-value***: Inconsistent p-values for direct and indirect comparisons in pairwise comparisons; ***Pooled p-value***: The combined p-value to assess the statistical significance of the overall effect.

Electronic Supplementary Material S9 (Sensitivity Analysis for Primary results)

Test Method	Condition	Hedges'g	95% CrI	SUCRA (%)	95% PI	Comparisons
**Exercise performance**						
r = 0.8	CAF	0.22	(0.05, 0.42)	0.25 (54%)	(-0.51, 0.95)	CAF-(CAF+SB): -0.07 (-0.15, 0.01)
r = 0.8	CAF+SB	0.29	(0.12, 0.49)	0.95 (91%)	(-0.43, 1.04)	CAF-SB: -0.004 (0.09, 0.08)
r = 0.8	SB	0.23	(0.05, 0.43)	0.30 (49%)	(-0.51, 0.98)	(CAF+SB)-SB: 0.07 (-0.02, 0.14)
r = 0.2	CAF	0.21	(0.02, 0.41)	0.39 (39%)	(-0.41, 0.83)	CAF-(CAF+SB): -0.06 (-0.22, 0.11)
r = 0.2	CAF+SB	0.27	(0.09, 0.47)	0.77 (64%)	(-0.34, 0.90)	CAF-SB: 0.01 (-0.16, 0.18)
r = 0.2	SB	0.20	(0.02, 0.40)	0.35 (48%)	(-0.41, 0.84)	(CAF+SB)-SB: 0.06 (-0.10, 0.23)
**Rating of perceived exertion**						
r = 0.8	CAF	-0.19	(-0.54, 0.16)	0.49 (86%)	(-1.06, 0.68)	CAF-(CAF+SB): 0.17 (-0.05, 0.38)
r = 0.8	CAF+SB	-0.36	(-0.72, -0.01)	0.97 (94%)	(-1.23, 0.51)	CAF-SB: -0.15 (-0.36, 0.05)
r = 0.8	SB	-0.04	(-0.39, 0.31)	0.04 (92%)	(-0.92, 0.82)	(CAF+SB)-SB: -0.32 (-0.54, 0.11)
r = 0.2	CAF	-0.19	(-0.58, 0.21)	0.50 (56%)	(-0.94, 0.57)	CAF-(CAF+SB): 0.16 (-0.27, 0.60)
r = 0.2	CAF+SB	-0.35	(-0.76, 0.05)	0.84 (74%)	(-1.11, 0.40)	CAF-SB: -0.15 (-0.57, 0.28)
r = 0.2	SB	-0.04	(-0.42, 0.34)	0.16 (73%)	(-0.78, 0.71)	(CAF+SB)-SB: -0.31 (-0.74, 0.13)
**Blood bicarbonate**						
r = 0.8	CAF	-1.14	(-2.07, -0.22)	1 (100%)	(-3.65, 1.32)	CAF-(CAF+SB): -0.96 (-1.27, -0.66)
r = 0.8	CAF+SB	-0.18	(-1.11, 0.75)	0.43 (86%)	(-2.65, 2.33)	CAF-SB: -1.11 (-1.42, -0.81)
r = 0.8	SB	-0.03	(-0.95, 0.89)	0.07 (86%)	(-2.50, 2.42)	(CAF+SB)-SB: -0.15 (-0.42, 0.13)
r = 0.2	CAF	-0.75	(-1.59, 0.02)	0.98 (96%)	(-2.71, 1.14)	CAF-(CAF+SB): -0.52 (-1.06, 0.05)
r = 0.2	CAF+SB	-0.23	(-1.02, 0.54)	0.37 (68%)	(-2.16, 1.73)	CAF-SB: -0.67 (-1.21, -0.14)
r = 0.2	SB	-0.09	(-0.87, 0.69)	0.15 (70%)	(-2.01, 1.82)	(CAF+SB)-SB: -0.14 (-0.65, 0.37)
**Blood pH**						
r = 0.8	CAF	-0.26	(-1.34, 0.81)	0 (0)	(-3.11, 2.53)	CAF-(CAF+SB): -0.93 (-1.22, -0.64)
r = 0.8	CAF+SB	0.67	(-0.41, 1.75)	0.50 (50%)	(-2.16, 3.52)	CAF-SB: -1.42 (-1.72, -1.12)
r = 0.8	SB	1.16	(0.08, 2.24)	1.00 (100%)	(-1.65, 3.94)	(CAF+SB)-SB: -0.49 (-0.77, -0.21)
r = 0.2	CAF	-0.25	(-1.04, 0.49)	0.004 (99%)	(-2.11, 1.54)	CAF-(CAF+SB): -0.66 (-1.21, -0.15)
r = 0.2	CAF+SB	0.39	(-0.35, 1.18)	0.52 (94%)	(-1.41, 2.27)	CAF-SB: -1.10 (-1.66, -0.54)
r = 0.2	SB	0.83	(0.09, 1.64)	0.97 (95%)	(-0.94, 2.72)	(CAF+SB)-SB: -0.44 (-0.98, 0.10)
**Blood lactate**						
r = 0.8	CAF	0.62	(-0.60, 1.75)	0 (100%)	(-3.00, 4.14)	CAF-(CAF+SB): -1.39 (-1.67, -1.11)
r = 0.8	CAF+SB	2.01	(0.78, 3.14)	1 (100%)	(-1.60, 5.61)	CAF-SB: -0.74 (-1.00, -0.48)
r = 0.8	SB	1.36	(0.14, 2.49)	0.50 (100%)	(-2.26, 4.86)	(CAF+SB)-SB: 0.65 (0.38, 0.93)
r = 0.2	CAF	0.45	(-0.45, 1.30)	0.005 (99%)	(-2.02, 2.87)	CAF-(CAF+SB): -1.19 (-1.73, -0.66)
r = 0.2	CAF+SB	1.64	(0.73, 2.53)	0.99 (99%)	(-0.82, 4.11)	CAF-SB: -0.59 (-1.10, -0.10)
r = 0.2	SB	1.04	(0.15, 1.90)	0.50 (98%)	(-1.42, 3.46)	(CAF+SB)-SB: 0.60 (0.08, 1.13)

Notes: r = correlation coefficient; SUCRA (%) = surface under the cumulative ranking curve (Probability of corresponding ranking).
